# Measuring Near-Infrared Spectroscopy Derived Cerebral Autoregulation in Neonates: From Research Tool Toward Bedside Multimodal Monitoring

**DOI:** 10.3389/fped.2018.00117

**Published:** 2018-05-14

**Authors:** Liesbeth Thewissen, Alexander Caicedo, Petra Lemmers, Frank Van Bel, Sabine Van Huffel, Gunnar Naulaers

**Affiliations:** ^1^Department of Neonatology, University Hospitals Leuven, Leuven, Belgium; ^2^Department of Development and Regeneration, KU Leuven, Leuven, Belgium; ^3^Department of Electrical Engineering, ESAT-Stadius, KU Leuven, Leuven, Belgium; ^4^Interuniversity Microelectronics Centre, Leuven, Belgium; ^5^Department of Neonatology, University Medical Center Utrecht, Utrecht, Netherlands

**Keywords:** cerebral autoregulation, NEAR-infrared spectroscopy (NIRS), neonate, cerebral blood flow (CBF), arterial blood pressure, outcome, multimodal monitoring, mathematical model

## Abstract

**Introduction:** Cerebral autoregulation (CAR), the ability of the human body to maintain cerebral blood flow (CBF) in a wide range of perfusion pressures, can be calculated by describing the relation between arterial blood pressure (ABP) and cerebral oxygen saturation measured by near-infrared spectroscopy (NIRS). In literature, disturbed CAR is described in different patient groups, using multiple measurement techniques and mathematical models. Furthermore, it is unclear to what extent cerebral pathology and outcome can be explained by impaired CAR.

**Aim and methods:** In order to summarize CAR studies using NIRS in neonates, a systematic review was performed in the PUBMED and EMBASE database. To provide a general overview of the clinical framework used to study CAR, the different preprocessing methods and mathematical models are described and explained. Furthermore, patient characteristics, definition of impaired CAR and the outcome according to this definition is described organized for the different patient groups.

**Results:** Forty-six articles were included in this review. Four patient groups were established: preterm infants during the transitional period, neonates receiving specific medication/treatment, neonates with congenital heart disease and neonates with hypoxic-ischemic encephalopathy (HIE) treated with therapeutic hypothermia. Correlation, coherence and transfer function (TF) gain are the mathematical models most frequently used to describe CAR. The definition of impaired CAR is depending on the mathematical model used. The incidence of intraventricular hemorrhage in preterm infants is the outcome variable most frequently correlated with impaired CAR. Hypotension, disease severity, dopamine treatment, injury on magnetic resonance imaging (MRI) and long term outcome are associated with impaired CAR. Prospective interventional studies are lacking in all research areas.

**Discussion and conclusion:** NIRS derived CAR measurement is an important research tool to improve knowledge about central hemodynamic fluctuations during the transitional period, cerebral pharmacodynamics of frequently used medication (sedatives-inotropes) and cerebral effects of specific therapies in neonatology. Uniformity regarding measurement techniques and mathematical models is needed. Multimodal monitoring databases of neonatal intensive care patients of multiple centers, together with identical outcome parameters are needed to compare different techniques and make progress in this field. Real-time bedside monitoring of CAR, together with conventional monitoring, seems a promising technique to improve individual patient care.

## Introduction

**Figure 1:**
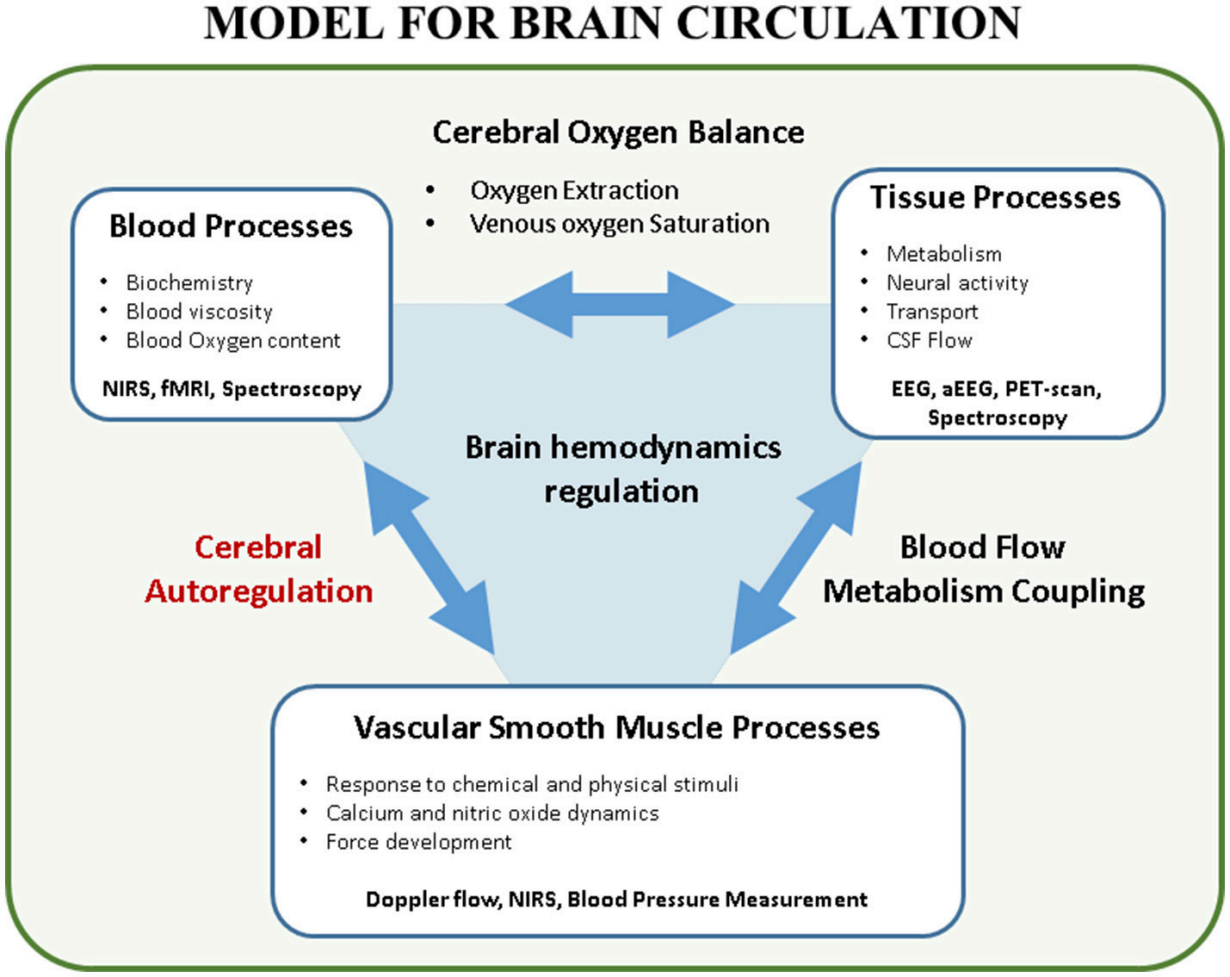
Model for brain circulation. Cerebral autoregulation is only a single element in the interaction between blood processes and vascular smooth muscle processes. Multiple factors play a role in brain hemodynamics regulation. This figure shows the complex interaction between the different processes in brain circulation. Furthermore, techniques to study these processes in a non-invasive way are reported (with permission (4)). EEG, (amplitude-integrated) electroencephalogram; CSF, cerebrospinal fluid; fMRI, functional magnetic resonance imaging; NIRS, near-infrared spectroscopy; PET, positron emission tomography.

The transitional period in neonates is an extremely vulnerable phase prone to hemodynamic instability (i.e., hypotension, cyanosis, shock, ischemia, reperfusion injury) at high risk for cerebral ischemic and/or hemorrhagic lesions. At the same moment interventions such as intubation, surfactant administration, cooling in hypoxic-ischemic encephalopathy (HIE), treatment with inotropes, surgery and sedation can increase or decrease this risk. Maintaining adequate brain tissue oxygenation, a stable balance between cerebral oxygen delivery and extraction, is one of the major goals in neonatology because brain damage by ischemic or hemorrhagic lesions will often lead to impaired neurodevelopmental outcome (1). Ninety percent of the intraventricular hemorrhages (IVH) occur in the first 72 h after birth, suggesting that brain circulation is especially vulnerable in this period (2). To monitor and improve brain tissue oxygenation, arterial blood pressure (ABP) is often used as a surrogate measurement for cerebral blood flow (CBF). However, this classical early-goal directed therapy -increasing ABP if below a cut-off value- may not be adequate nor ideal in the healthy preterm or sick neonate in the transitional phase (3). Several mechanisms play a role in maintaining cerebral oxygenation, ABP being only one factor in this very complicated physiological system (4) (Figure 1, with permission). In this introduction, the determinants of cerebral oxygen delivery are discussed. Then, the concept of static and dynamic cerebral autoregulation (CAR) is explained with an emphasis on its assessment using near-infrared spectroscopy (NIRS).

Cerebral oxygen delivery is determined by CBF and blood oxygen content. CBF itself is the result of the gradient between cerebral perfusion pressure (CPP) and cerebrovascular resistance (CVR). CPP is determined by ABP and intracranial pressure (ICP).

(1) $$\begin{array}{l} \text{CBF}=\frac{\text{CPP}}{\text{CVR}}=\frac{\text{ABP}-\text{ICP}}{\text{CVR}} \end{array}$$

CVR reflects the varying tone of smooth muscle cells in the wall of arteries. One of the important factors influencing this smooth muscle tone is ABP. The myogenic reflex will cause the vessel to constrict or dilate if CPP increases or decreases, respectively. This reflex leads to the classical concept of CAR, the mechanism in which CBF is maintained stable regardless of changes in ICP and first described in humans by Lassen (5). If ICP is stable, CPP can be replaced by ABP. In this way, changes in CBF can be measured for a range of ABP values to determine CAR. However, multiple factors, apart from changes in ABP, can influence smooth muscle tone. The most relevant is the chemical influence of pCO_2_ on the muscle tone, known as CO_2_ vaso-reactivity, but also effects of NO, calcium and physical stimuli have been described (6, 7). Furthermore, different tissue processes (i.e., functional activation, autonomic neural activity, among others) cause alteration in regional CBF. This is known as blood flow metabolism coupling and is more extensively described in the paper by Huneau et al. (8). In this review article we will focus on the classical definition of flow-pressure CAR.

A good overview of the first flow-pressure CAR studies is provided by Weindling et al and Greisen (9, 10). In this review it is indicated that *static* CAR can be studied as a steady-state response: looking at the relationship between CBF and CPP (ABP) without considering the time course of changes in flow following changes in pressure. Impaired CAR (CBF being strongly correlated with ABP) in preterm infants is described using ^133^Xe clearance or plethysmography after which it was hypothesized that loss of CAR plays a decisive part in the pathogenesis of brain lesions in the neonate (11–14). To study *dynamic* flow-pressure CAR, the continuous measurement of changes in CPP, and thus ABP, is mandatory. Spontaneous rhythmic oscillations in ABP and CBF are used to describe the CAR mechanisms within different frequency bands. The low and very low frequency bands are dominated by myogenic, neurogenic and metabolic regulatory factors. Sometimes, the lack of a large variation in CPP and CBF makes it necessary to challenge the regulatory mechanisms in order to be able to measure autoregulation reliably. Therefore, some studies have induced changes in ABP (15) while other use the changes in ABP due to pathology, in order to be able to assess CAR. This might seem as a limiting factor for the continuous monitoring of CAR. However, the more stable the clinical situation, the less problems regarding CAR are expected.

The dynamical aspect of CAR can be measured using Doppler flowmetry or NIRS. Doppler flowmetry uses ultrasound to measure blood flow velocity in a specific vessel, measuring the immediate effect of changes in blood pressure on the CBF velocity, while NIRS reflects the effect of changes in ABP over a longer period of time. For a further review of the Doppler technique to measure CAR, we refer to Panerai et al. (16). With NIRS, cerebral oxygen saturation and cerebral fractional tissue oxygen extraction (cFTOE) can be measured (17, 18). NIRS is based on the specific absorption of near-infrared light by oxyhemoglobin (HbO_2_) and deoxyhemoglobin (HHb) in cerebral tissue, reflecting the mixed venous-capillary-arterial oxygenation state of the parenchyma directly beneath the light emitting sensor. It has been shown that under constant arterial oxygen saturation (SaO_2_) and a constant brain metabolism, NIRS derived cerebral oxygen saturation can be used as a surrogate measurement for CBF. This was first described by Tyszczuk et al, describing normal CBF even during periods of low ABP (19).

The use of NIRS to study CAR was first documented by Tsuji et al, using the correlation between the hemoglobin difference (HbD) and mean ABP, in periods of stable oxygen saturation, in preterm infants. They were the first to correlate impaired CAR with brain lesions, mainly IVH, using the classical NIRS technology with the Beer-Lambert law (20). The introduction of spatially resolved spectroscopy, using different receptors in the light emitting sensor, led to measurements less prone to movement artifacts and thus more easy to use in clinical situations. Wong et al. were the first to describe impaired CAR, assessed by means of the brain tissue oxygenation index (TOI) in the sickest infants with the highest clinical risk index for babies (CRIB) scores (21).

The dynamic approach might become the reference method for clinical assessment of CAR. However, the major disadvantage is the lack of standardization in the measurement. The dynamics in time and magnitude between changes in ABP and NIRS derived cerebral oxygenation can be assessed by different mathematical models. These models range from the simple time-domain correlations between cerebral oxygenation and ABP, which assumes autoregulation is a simple linear process, to more complex techniques, based on continuous wavelet transforms (CWT), to describe the stochastic, non-stationary dynamic nature of CAR.

Since measurement of cerebral oxygenation is easy and non-invasive in neonates, several groups have been studying the added value of measuring CAR in different patient groups, using different NIRS instruments, different mathematical models and different outcome parameters in the last 2 decades. The aim of this review is to provide a general overview of the clinical framework to study CAR by describing and explaining the different preprocessing methods and mathematical models. Furthermore, we will critically summarize the available literature.

## Methods

Between July 2015 and 20 November 2017, we conducted an extensive search of the literature to identify clinical studies measuring NIRS-derived CAR in neonates. This search was conducted using the PRISMA checklist (http://prisma-statement.org) in the PUBMED and EMBASE database. The search strategy included the terms (cerebrum OR cerebral OR brain) AND (homeostasis OR autoregulation) AND (infant, newborn OR infant OR newborn infant OR neonates) AND (spectroscopy, near-infrared OR spectroscopy AND near-infrared OR NIRS OR near infrared spectroscopy OR near AND infrared AND spectroscopy) without language restriction. Inclusion criteria were (1) clinical study in (2) neonates measuring quantitatively (3) flow-pressure CAR using (4) NIRS methodology. We excluded studies or parts of certain methods/results measuring other parameters (i.e., heart rate, cardiac output) to evaluate CAR. Also, abstracts only, case reports, and articles limited to methodological questions without short or long term outcome were excluded. LT screened the article titles and abstracts to determine whether they met the inclusion criteria. Then, LT reviewed full text articles to assess for eligibility. Any articles presenting doubts or inconsistencies were fully reviewed by GN and AC until a decision was reached on their inclusion or exclusion. By using cross-references, additional eligible articles were added by hand searching.

We collected study type and data about patient numbers, gestational age (GA), postmenstrual age (PMA) or day of life (DOL) at start of the study. Furthermore, data about the mathematical model, initial sample frequency of data extraction, epoch length and duration of measurement per patient was extracted. The proposed definition of impaired CAR and the outcome determined by the authors was defined. Different patient groups according to pathology were identified to summarize the results.

## Results

**Figure 2:**
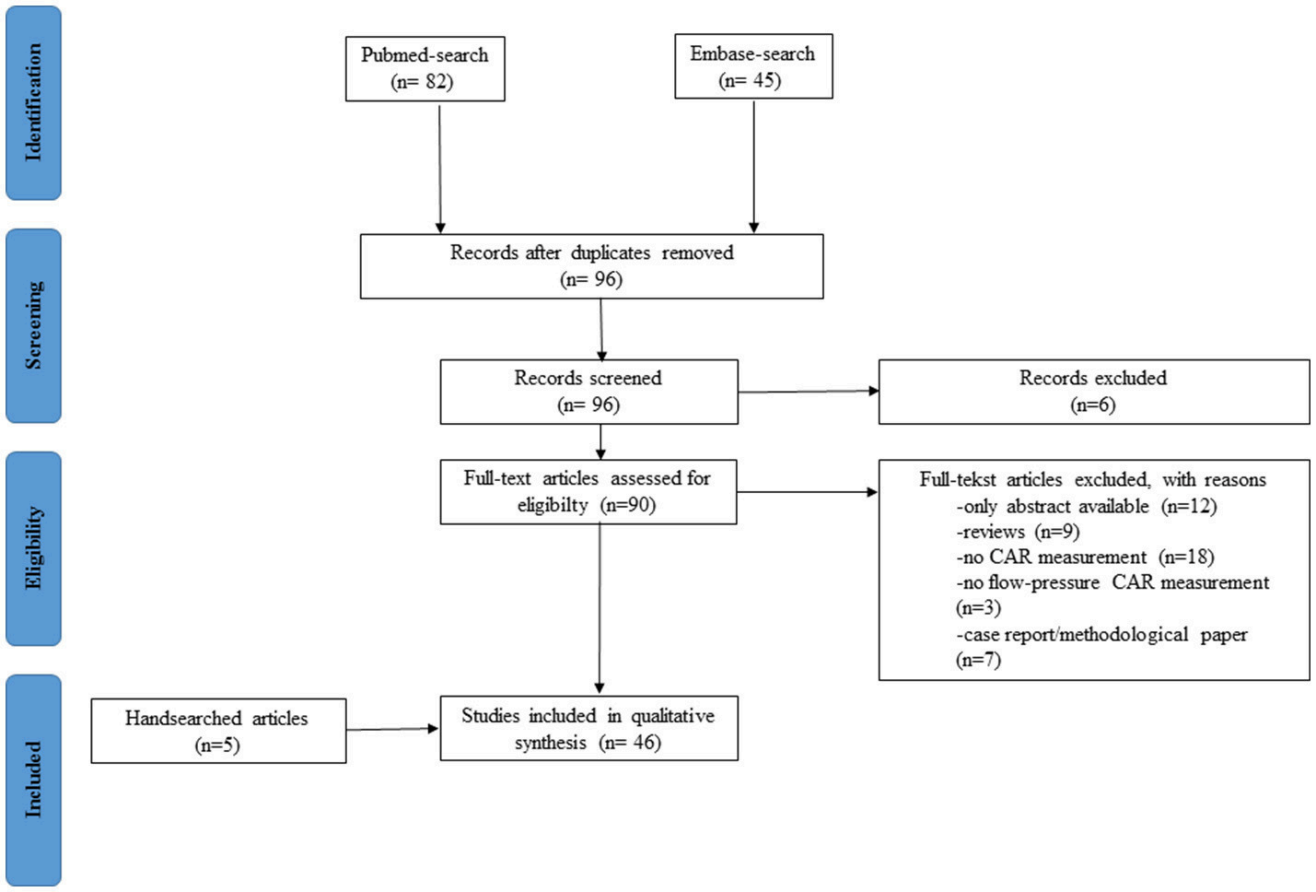
Adapted PRISMA flow diagram.

The result of the systematic search is presented in an adapted PRISMA flow diagram (Figure 2). Included studies are organized in 4 different patient groups: (1) preterm infants during the transitional period, (2) neonates receiving medication/treatment, (3) neonates with congenital heart diseases (CHD), and (4) neonates with HIE treated with therapeutic hypothermia. An overview of the different studies per patient group is provided in Table 1.

**Table 1:** Overview of included studies.

**Author**	**-Patient number** -GA/PMA in weeks in mean (SD) or median (range)/^*^DOL **-Study type**	**NIRS instrument**	**-Mathematical model** **-Sample frequency** **-Epoch length** **-Mean or median duration**	**Definition impaired CAR**	**Outcome**
**PRETERMS DURING THE TRANSITIONAL PERIOD**					
Schat et al. (26)	-*n =* 28 -case: 27.9 (26.3–34.7); control: 27.4 (25.6–34.7) -Case control	INVOS 5100C	-COR between MAP and FTOE -0.003 Hz -5 min -48 h	Significant negative COR coefficient	No COR between presence of impaired CAR and NEC
Riera et al. (34)	-*n* = 54 -27 (1.9) -Cohort	NIRO-200nx	-(p)PD COH between MAP TOI in 0.003–0.04 Hz band -2 Hz -30 min -9.5 h	-Threshold PDC_MAP>>TOI_ for low SVC flow was 0.554 = PDC_MAP>>TOI_ classifier -Low SVC flow as surrogate for cerebral hypoperfusion	-PDC_MAP>>TOI_ predicted low SVC flow -PDC_MAP>>TOI_ classifier associated with % of time of MABP <GA-5 mmHg -pPDC _MAP>>TOI_ predicted cardiovascular support, severe IVH and death
Vesoulis et al. (69)	-*n* = 62 -25.4 (1.3) -Cohort	Foresight	-Log transformation of TF gain between MAP and SctO_2_ in 0.08–0.12 Hz band -0.5 Hz -20 min -68 h	Stronger dampening (more negative TF gain coefficient) is better autoregulation	-Greater dampening independently associated with advancing GA, BW and chorioamnionitis -Less dampening independently associated with African-American race, IVH during first week
Stammwitz et al. (70)	-*n* = 31 -27^2/7^ (26^1/7^-32^2/7^) -Cohort	Critikon Cerebral Oxygenation Monitor 2001	-COH between MAP and tHb/OI in 0–0.01 Hz band -2 Hz -≥12 min -8 h	No cut off. Hypothesis that high COH indicate a coordination of physiological sub-systems and thus are a sign of health	Low COH in the first 24 h were associated with IVH ≥ 3, death and MDI
Binder-Heschl et al. (39)	-*n* = 46 -case: 33.4 (1.9); control: 33.3 (1.3) -Case control	INVOS 5100 C	-COR between (invasive *n* = 3) MABP and crSO_2_ -0.13 Hz -1 h -24 h	No definition	Very weak COR between MABP and crSO2 suggesting intact CAR during borderline hypotension
Eriksen et al. (67)	-*n* = 60 -26.6 (1.3) -Cohort	NIRO 300	-COx (moving linear COR), regression coefficient vs. COH, TF gain between MAP and OI in 0.003–0.04 Hz band -2 Hz -10 min -2.3 h	COx ≥ 0.4 and COH ≥ 0.5	-COR between TF gain and regression coefficient was weak (*r* = 0.245) but significant after exclusion of outliers -COx is more robust, TF gain also increases if MAP and OI are in counterphase
Riera et al. (72)	-*n* = 54 -27 (1.9) -Cohort	NIRO 200 NX	-(p)BiAR-COH, (p)COH between MABP and TOI in 0.003–0.04 Hz band -2 Hz -30 min -9.5 h	-Threshold BiAR-COH and COH for low SVC flow was 0.58 and 0.52 respectively -Low SVC flow as surrogate for cerebral hypoperfusion	-BiAR-COH better in predicting low SVC flow with compared to COH, -pBiAR-COH but not pCOH was associated with IVH grade 3–4 and predicted mortality
Verhagen et al. (30)	-*n* = 25 -29.1 (25.4–31.7) -Cohort	INVOS 4100–5100	-COR between MABP and r_c_SO_2_/FTOE -0.003 Hz -5 min -24 h	Statistically significant positive and negative COR between r_c_SO_2_-MABP and FTOE/MABP, respectively	Identification of absent CAR in 40% of patients, no correlations between absent CAR and clinical variables except higher hemoglobin levels
Caicedo et al. (71)	-*n* = 9 - <32 -Case control	INVOS 4100–5100	-BPRSA between MABP and rScO_2_ -1 Hz -? -?	No definition	Presence of non-linear relations between the variables. In addition, the BPRSA curves from the control subjects converge faster to zero than the curves for the subjects with IVH gr 3–4.
Alderliesten et al. (42)	-*n* = 90 -24^6/7^-31 -Case control	INVOS 4100–5100	-COR between MABP and rScO_2_ -1 Hz -1 min -Cases: 48 h; controls: 47 h	COR > 0.5	-More time with impaired autoregulation before and after detection of PIVH compared to controls
Hahn et al. (66)	-*n* = 60 -27 (1.3) -Cohort	NIRO 300	-COH, TF gain between MAP and OI in 0.003–0.04 and 0.04–0.1 Hz band -2 Hz -10 min -2.3 h	COH ≥ 0.45–0.47	-Negative association between TF gain and MAP -No association between impaired CAR and antenatal or postnatal signs of inflammation, IVH or mortality
Caicedo et al. (63)	-*n* = 42 -28.1 (2.27) -Cohort	Criticon Cerebral RedOx monitor	-TF gain, phase between MABP and HbD in 0.003–0.02, 0.02–0.05 and 0.05–0.1 Hz band -0.333 Hz -20 min -72 h	No definition	-Significant higher TF gain in normal compared to abnormal (IVH, PVL, death, abnormal MDI and/or PDI) population in 0.05–0.1 Hz band
Wong et al. (73)	-*n* = 32 -26.3 (1.5) -Cohort	NIRO 200	-COH and TF gain between MABP and TOI in 0.003–0.02 Hz band -6 Hz -20 min -5 × 20 min on day 1-2-3	COH ≥0.5	-High COH and TF gain at low BPV in unstable children with brain injury. -Significant association between maximum COH and BPV in stable children.
Zhang et al. (60)	-*n* = 17 -26 (2) -Cohort	NIRO 300	-COH, TF gain and phase between MAP and HbO_2_/HHb/HbD/TOI in 0.02–0.04, 0.04–0.15, 0.15–0.25 Hz band -1 kHz -10 min -10 min	COH ≥ 0.5 or max COH if <0.5	-Multiple testing -Strongest relation was found between COH MAP-HHb in 0.04–0.15 Hz band and CRIB-II
Caicedo et al. (57)	-*n* = 33 and 20 -28.9 (1.8) and 28.4 (3.5) -Cohort	NIRO 300 and INVOS 4100	-COR, (PA)COH, between MABP and HbD/TOI/rSO_2_ in 0.003–0.1 Hz band -0.333 Hz -20 min -50–70 and 6–9 h	COR/(PA)COH>0.5 CPRT COR/(PA)COH: % time with impaired CAR	No significant correlation with CRIB/MDI/PDI/Griffith score and mean or CPRT COR/(PA)COH
Gilmore et al. (44)	-*n* = 23 -26.7 (1.4) -Cohort	Foresight	-COx (moving linear COR) between MABP and SctO_2_ -0.5 Hz -5 min -3.2 days	COx > 0.5	Impaired autoregulation was associated with low MABP but not with IVH.
Hahn et al. (56)	-*n* = 22 -27.5 (24.1–29.4) -Cohort	NIRO 300	-COH between MAP and OI in 0.003–0.04 and 0.04–0.1 Hz band -2 Hz -10 min -2.1 h	Threshold COH with simulation COH_ST_ = COH minus ThresholdCOH COH_ST_ ≥ 0 implies impaired CAR	-Precision of COH to measure CAR is improved when the magnitude of variability in ABP is taken into account
De Smet et al. (32)	-*n* = 10, 10 and 10 -28^1/7^ (2^1/7^), 29^2/7^(1^2/7^) and 28^5/7^ (3^2/7^) -Cohort	Critikon Cerebral Oxygentation monitor 2001, INVOS 4100 and NIRO 300	-(PA)COH between MABP and HbD/rSO_2_/TOI in 0.0033–0.04 Hz band -1.677, 1 and 10 Hz -10.15 and 12.5 min -72 h	(PA)COH>0.5 PPI: % epochs with impaired CAR CPRT: % time with impaired CAR	-High PCOH values are better indicators of poor clinical outcome (MDI <84, PDI <84, Apgar <7) than COH -CPRT and PPI are better indicators of poor clinical outcome than mean score values
O'Leary et al. (64)	-*n* = 88 -26 (23–30) -Cohort	NIRO 500	-COH, TF gain between MAP and HbD in 0.05–0.25, 0.25–0.5 and 0.5–1.0 Hz bands -2 Hz -10 min -75.2 h	COH > 0.69	High TF gain was significantly associated with IVH in 0.05–0.25 Hz band
De Smet et al. (40)	-*n* = 20 -28.7 (24–39) -Cohort	NIRO 300	-COR, (PA)COH between MAP and HbD/TOI in 0–0.01 Hz band -0.2 Hz -30 min -?	COR, (PA)COH > 0.5 CPRT: % time with impaired CAR	-TOI may be used for the calculation of cerebral autoregulation. -CPRT generates a measurement of the autoregulation impairment proportional to COR and PA(COH)
Wong et al. (21)	-*n* = 24 -26 (2.3) -Cohort	NIRO 300	-COH, TF gain between MAP and TOI in 0.003–0.02, 0.02–0.05, 0.05–0.1 Hz band -1 Hz -20 min -52 min	COH ≥ 0.5	-High COH and high TF gain were found in sickest infants in 0.003–0.02 Hz band -CRIB score best predictor of COH -COH ≥ 0.5 predictive for mortality
Soul et al. (59)	-*n* = 90 -26.5 (23–30) -Cohort	NIRO 500	-COH between MAP and HbD in 0–0.04 Hz band -2 Hz -10 min -17.2 h	-COH ≥0.77 -PPI: % epochs with impaired CAR	-Pressure passive cerebral circulation associated with GA and BW, hypotension, maternal hemodynamic factors. -Pressure passivity in 87/90 patients, with mean PPI of 20.3% (range 0–48.6)
Lemmers et al. (29)	-*n* = 38 -case 28.6 (1.32); control 29.3 (1.74) -Case control	INVOS 4100	-COR between MAPB and ScO_2_/FTOE -10 Hz -15 min -420 min	-COR MABP/ScO_2_ > 0.5 -COR MABP/FTOE <-0.5	More 15 min periods with impaired autoregulation in RDS in comparison with no RDS
Morren et al. (65)	-*n* = ? -? -Cohort	NIRO 300	-COR, COH, CPC between MAP and HbD in 0–0.01 Hz band -0.2 Hz -30 min -?	No definition	CPC and COR are better measures to detect impaired autoregulation than COH analysis.
Tsuji et al. (20)	-*n* = 32 -27.1 (2.5) -Cohort	NIRO 500	-COH between MAP and HbD in 0–0.01, 0.01–0.05 and 0.05–0.1 Hz band -2 Hz -30 min -207 min	COH > 0.5	-Impaired autoregulation in 0–0.01 Hz band was observed in 53% of patients and in 80% of patients with IVH grade 3/4 or PVL
**NEONATES RECEIVING MEDICATION/TREATMENT**					
Li et al. (75)	-*n* = 44 -case:29.5 (1.3); control: 29.3 (1.6) -Case control	MC-2030C Cerebral oximeter	-COR between MAP and ScO_2_ -0.1 Hz -5 min -20 min	Deviation from baseline of COR coefficient suggests less effective CAR	Longer lasting impaired CAR with surfactant administration with INSURE compared to LISA method
Alderliesten et al. (74)	-*n* = 132 -case: 29^2/7^ (25^6/7^-31^4/7^); control: 29^3/7^ (25^5/7^-31^4/7^) -Case control	INVOS 4100–5100	-COR between MABP and rScO_2_ -1 Hz -15 min -72 h	% time with COR > 0.5	Impaired CAR associated with treatment with higher doses of dopamine compared to no blood pressure support
Eriksen et al. (45)	-*n* = 60 -case: 26.2(1.5); control: 26.7(1.2) -Case control	NIRO 300	-COx (moving COR) between MAP and OI -2 Hz -10 min -2.3 h	COx > 0	Impaired CAR associated with dopamine treatment compared to no dopamine treatment
Baerts et al. (58)	-*n* = 18 -case: 27.2 (25^2/7^-29^4/7^) control: 27.4 (25^0/7^-29^5/7^) -Case control	INVOS 4100–5100	-COR in the very slow frequency range (1/60 HZ) between MABP and rScO_2_ -1 Hz -15 min -1 h	COR > 0.5 during 10% or more of time	No difference in CAR between offsprings of mothers treated with indomethacine and controls
Caicedo et al. (62)	-*n* = 56 -29 (24.7–31.9) -Case control	INVOS 4100	-COR, COH and TF gain between MABP and rScO_2_ in 0.003–0.02, 0.02–0.05 and 0.05–0.1 Hz band -1 Hz -15 min -72 h	High TF gain	Higher TF gain in offsprings of mothers treated with labetalol during 1 day of life in 0.003–0.02 and 0.02–0.05 Hz band compared to controls
Kooi et al. (31)	-*n* = 14 -26.7 w -Cohort	INVOS 5100 C	-COR between changes in MABP and cFTOE -? -10–30 min -2 h	Increase in MABP of 2 mmHg combined with decrease of cFTOE of 5%	Unable to define subgroup of infants lacking CAR after volume treatment
Papademetriou et al. (24)	-*n* = 6 -^*^day 3–16 -Interventional	Hitachi ETG-100	-CWT, WCC between MAP and HbO_2_ in 0.06–0.13, 0.13–0.25 and 0.25–1 Hz band -5 Hz -10 min -70 min	WCC > 0.5	-Loss of CAR at low ECMO flow -Right hemisphere more susceptible to low flow
Chock et al. (41)	-*n* = 40 -26 (1) -Case control	INVOS 5100	-COR between MAP and rSo_2_ -0.2 Hz -20 min -26 h	-COR > 0.5 -PPI:% epochs with impaired CAR -max COR	-PPI was significantly higher 2 h after ductal ligation compared with control and indomethacin PDA treatment -Dopamine use was associated with max COR, independent of PDA treatment strategy
Wagner et al. (15)	-*n* = 24 (11 neonates) -neonates >36 weeks; other 2 month-15 year -Interventional	NIRO 500	-$\frac{\text{ΔH}{\text{b}}_{\text{Diff}}}{\text{ΔMABP}}$, $\frac{\text{ΔH}{\text{b}}_{\text{Total}}}{\text{ΔMABP}}$ after phenylephrine bolus -2 Hz -5–60 s -240 s	ARI_HbDiff_/ARI_HbTotal_ >0	-Significant correlations between ARI using cerebral HB signals and direct CBF measures -Studying dynamic CAR using bolus phenylephrine is faster and has better signal-to-noise ratio compared to use of spontaneous blood pressure fluctuations
Munro et al. (53)	- *n* = 17 -26 (0.4) -Case control	NIRO 500	-Linear regression of CBF vs. MAP -? -30 min -4 short measurements	Identification of breakpoint of MAP at which the residual sums of squares reaches a minimum	-CBF is autoregulated above 29 mmHg in extreme prematures -No evidence of autoregulation in dopamine treated infants
**NEONATES WITH CONGENITAL HEART DISEASE**					
Smith et al. (51)	-*n* = 64 -^*^day 9 (4–30) -Cohort	Reflectance spectroscopy monitor	-HVx (moving COR) between ABP and blood volume index -240 Hz -300 s -414 min	COR > 0	-Hypothermia was associated with hypotension, dysautoregulation and increased cerebral oximetry but collinearity between 3 variables during CPB
Votava-Smith et al. (76)	-*n* = 24 -38.6 (0.9) -Cohort	FORE-SIGHT	-COH between MAP and S_ct_O_2_ in 0.003–0.04 Hz band -0.5 Hz -20 min -23.4 h	COH > 0.58 % of epochs/patient with abnormal CAR	-All subjects had epochs with impaired CAR, with mean 15.3% (3.5–56%) during first days of life -Lack of sedative medication, low hemoglobin, low MAP and greater FTOE were associated with impaired CAR
Brady et al. (43)	-*n* = 54 -^*^25 months (0–222) -Cohort	INVOS	-COx (moving COR) between MABP and rSO_2_ -60 Hz-0.25 Hz -300 s -?	COx > 0.4, sorting by MABP, to determine LLA	-Broad range of individual LLA during CPB -CPB was associated with both hypotension and impairment of CAR
Bassan et al. (55)	-*n* = 43 -^*^day 9 (2–210) -Cohort	NIRO 500	-COH between ΔMAP and ΔHbD in 0–0.1 Hz band -2 Hz -5 min -105 min	-COH > 0.5 -PPI: % epochs with impaired CAR	In early postoperative phase after CPB, higher end- tidal CO_2_ and higher MAP variability increased odds of impaired CAR
**NEONATES WITH HYPOXIC-ISCHEMIC ENCEPHALOPATHY TREATED WITH THERAPEUTIC HYPOTHERMIA**					
Chavez-Valdez et al. (47)	-*n* = 75 -38^6/7^ (1^6/7^) -Cohort	INVOS 5100	-HVx (moving COR) between MAP and rTHb -100 Hz -300 s -57.8 h	-HVx>0 -% time, max deviation and AUC below MAP_opt_	Impaired CAR in HIE and therapeutic hypothermia correlated with cardiopulmonary injury and sex
Lee et al. (77)	-*n* = 64 -^*^day 1 -Cohort	INVOS 5100	-HVx (moving COR) between MAP and rTHb -100 Hz -300 s -58.1 h	-HVx > 0 -% time, max deviation and AUC below MAP_opt_	Impaired CAR during and after therapeutic hypothermia correlated with neurologic injury on MRI
Tian et al. (25)	-*n* = 9 -39(2) -Cohort	INVOS 4100–5100	-CWT between MAP and S_ct_O_2_ -0.033 Hz -not applicable - <72 h	Significant in-phase and anti-phase coherence between blood pressure and S_ct_O_2_	Impaired CAR correlated with MRI severity score and clinical outcome
Tekes et al. (48)	-*n* = 27 ->35 w, ^*^day 1 -Cohort	INVOS 5100	-HVx (moving COR) between MAP and rTHb -100 Hz -300 s -43 h	-HVx>0 -% time, max deviation and AUC below MAP_opt_	Impaired CAR during hypothermia and rewarming correlated with ADC scalars in specific anatomic regions on MRI
Massaro et al. (68)	-*n* = 36 -38.6 (1.7) and 39.2 (1.3) -Cohort	NIRO 200	-COH, TF gain between MAP and HbD in 0.05–0.25 Hz band -1 kHz -10 min -73 h	COH>0.384 PPI: % epochs COH>0.384 TF gain within pressure-passive epochs	Impaired CAR during hypothermia and rewarming correlated with MRI severity score or death
Burton et al. (49)	-*n* = 19 -38.9 (1.5) -Cohort	INVOS 5100	-HVx (moving COR) between MAP and rTHb -100 Hz -300 s -43 h	-HVx>0 -% time, max deviation and AUC below MAP_opt_	Impaired CAR during rewarming correlated with 2-year neurodevelopmental outcome
Howlett et al. (50)	-*n* = 24 -39.2(1.5) -Cohort	INVOS 5100	-HVx (moving COR) between MAP and rTHb -100 Hz -300 s -43.4 h	-HVx>0 -% time and max deviation below MAP_opt_	Impaired CAR during rewarming correlated with MRI injury severity in specific anatomic regions

First, we will present the clinical framework to study CAR by describing the different preprocessing methods and mathematical models, based on the literature search and identified clinical studies. Secondly, the definition of impaired CAR is discussed. Finally, the different study outcomes organized by the 4 identified patient groups are summarized.

### Clinical framework

**Figure 3:**
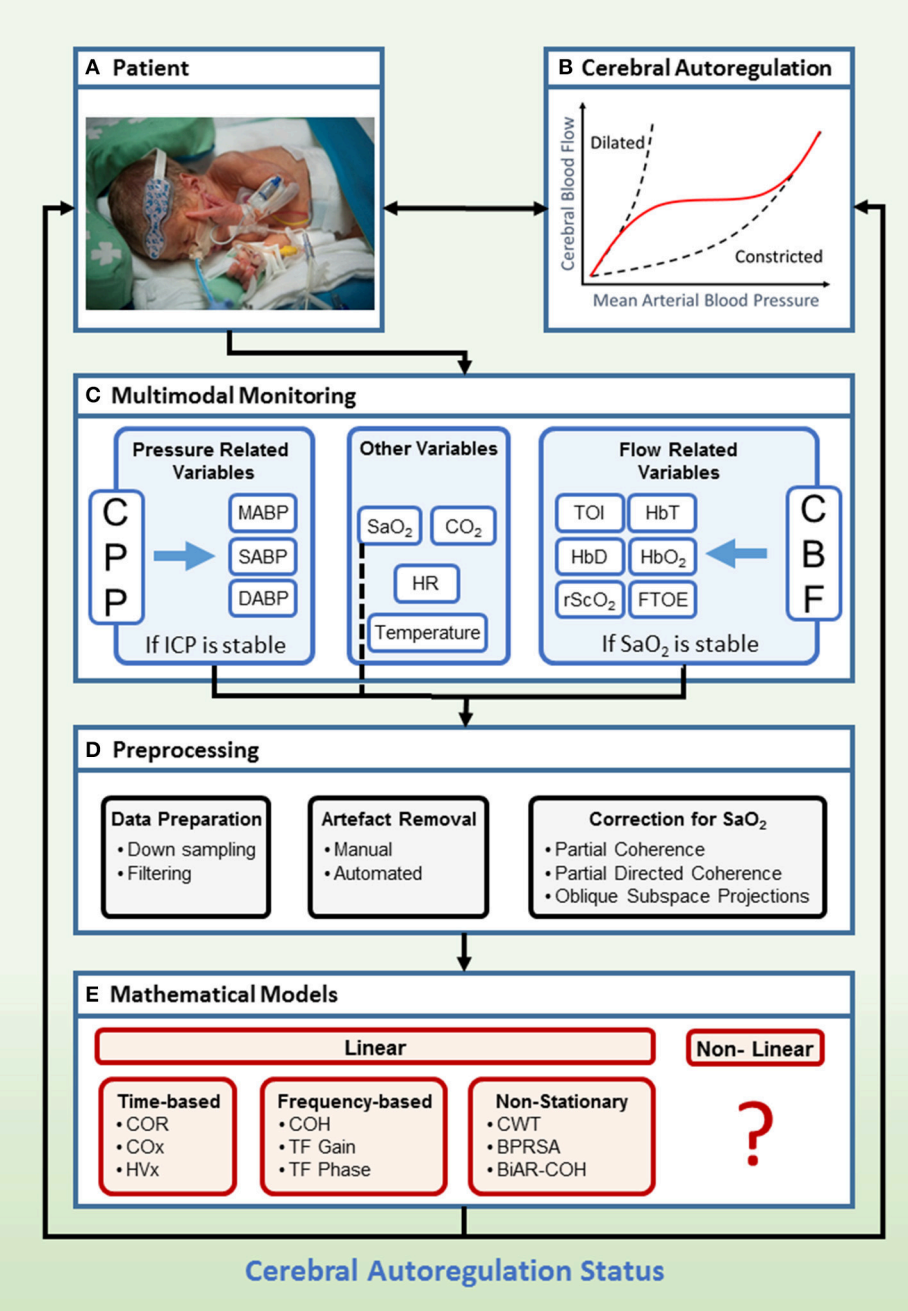
Clinical framework to study cerebral flow-pressure autoregulation status using multimodal monitoring. Hereby we propose a setup for determination of flow-pressure CAR in a NICU patient with typical age-appropriate monitoring **(A,B)**. In a multimodal setup **(C)**, invasive ABP and non-invasive vital parameters (SaO_2_, HR, CO_2_, Temperature), combined with non-invasive measurement of cerebral oxygenation, are collected continuously in a time-stamped method. If ICP is stable, ABP is a surrogate measurement for CPP. If SaO_2_ is stable, NIRS derived cerebral oxygenation is a surrogate measurement for CBF. The next step is preprocessing of the data, where the data is down sampled and filtered. Artifact removal and correction for SaO_2_ is applied **(D)**. Afterwards, several mathematical models can be applied **(E)**. The derived scores provide information about the status of the CAR mechanisms in the patient. Currently, analysis is done offline but real-time bedside information about the CAR status of the patient might be of interest to adapt treatment (written informed parental consent was obtained for publication of this image). BiAR-COH, bivariate autoregressive spectral coherence; BPRSA, bivariate phase rectified signal averaging; CAR, cerebral autoregulation; CBF, cerebral blood flow; CO_2_, carbon dioxide; COH, coherence; COR, correlation; COx, cerebral oximetry index; CPP, cerebral perfusion pressure; CWT, continuous wavelet transform; FTOE, fractional tissue oxygen extraction; HbD, hemoglobin difference; HbO_2_, ogygenated hemoglobin; HbT, total hemoglobin; HR, heart rate; HVx, hemoglobin volume index; ICP, intracranial pressure; M/S/DABP, mean/systolic/diastolic arterial blood pressure; NICU, neonatal intensive care unit; NIRS, near-infrared spectroscopy; rScO_2_, regional cerebral tissue oxygen saturation; SaO_2_, arterial oxygen saturation; TF, transfer function; TOI, tissue oxygenation index.

The clinical framework with general setup for the assessment of CAR is presented in Figure 3. The patient is connected to different monitors in order to acquire the relevant data for CAR assessment (Figure 3A). The main goal of this monitoring system is to indicate the status of the CAR mechanisms (Figure 3B). Recent advances enable the use of multimodal monitoring technologies, where different signals can be acquired at the same time (Figure 3C). In this context, different surrogates for CPP and CBF can be obtained. Additionally, other systemic parameters, which may influence CAR assessment can be measured. When the signals are obtained, different preprocessing algorithms are used in order to retrieve a reliable assessment of CAR (Figure 3D). Once the data are ready to be processed, different mathematical models exist in order to assess CAR (Figure 3E). The output from these models is then confronted to the state of the neonate, and its prognostic value is assessed. In the following sections we will describe the preprocessing methods and mathematical models more in detail.

#### Preprocessing

##### Data preparation

Different methods exist to transfer the requested parameters from the bedside monitor to an off-line system for processing. Even with different manufacturers, similar processing of the often large data files is necessary. To compare studies, detailed description of the different steps used to transfer the data is mandatory. Data is acquired with a given sampling frequency f_s._ The sampling frequency is extremely variable in the different studies and ranges from 100 to 0.03 Hz (Table 1).

Different filtering techniques are applied to the data. Generally, low-pass filters are used to remove high frequency oscillations. It is particularly common that the systemic data and the NIRS data are acquired at different sampling frequencies. In those cases, data is normally filtered with an anti-aliasing filter and down sampled in order to have a common sampling frequency of all the measurements. Different studies report different final sampling frequency values (22–24).

##### Artifact removal

When overlooking large data files, sudden, non-physiological changes in baseline or excessive variance can be due to artifacts. The main source of artifacts in the ABP can be due to disconnection of the sensor or blood withdrawal from the arterial catheter. In the case of NIRS, the main source of artifacts is due to the movement or replacement of the sensor, which causes a sudden offset for the measurements. Visual or automated artifact removal is possible and both methods are described in the different studies. However, due to the multifactorial nature of artifacts, automated methods are mostly accompanied by visual inspection. Once artifacts have been detected, they can be corrected by linear interpolation (25) or simply eliminated (20, 26) for further analysis. An example of automated artifact removal is proposed by Scholkmann et al. This method uses a moving window and detects the artifact by detecting sudden changes in the standard deviation of the signals, and corrects it by using spline interpolation of the affected segment (27). Other methods using moving windows are also described (28).

##### Correction for SaO_2_

Arterial oxygen saturation (SaO_2_) has a major influence on NIRS derived cerebral oxygenation, leading to a hypoxic (low oxygen content) cerebral desaturation but not necessarily an ischemic (low blood flow) cerebral desaturation. Different methods have been used in order to correct for the influence of third variables, such as the variability in SaO_2_. Most prevalent is the exclusion of data with variability in SaO_2_ larger than 5%. Therefore, conclusions are based on patients during stable SaO_2_ and extrapolation toward impairment in CAR during desaturation episodes is not possible. Several groups have used cFTOE instead of brain oxygenation to correct for changes in SaO_2_ (26, 29–31). However, whether this is a valid measurement technique is yet to be defined. De Smet et al. proposed the use of partial coherence (PACOH) in order to correct for variations in SaO_2_ on the NIRS signals (32). Another technique, partial directed coherence (PDC) has been developed by Baccala et al. to take out the influence of a third signal (33). Although used by Riera et al., this correction was not applied since they excluded the SaO_2_ signals in their analysis (34). Caicedo et al. proposed the use of oblique sub-space projections (ObSP) (35, 36). ObSP makes use of sub-space system identification that uses input-output observations of the system in order to produce a mathematical model that can explain the measured output. Furthermore, ObSP is able to decouple the linked dynamics between the different underlying subsystems in order to decompose the observed output in terms of the partial contributions of each input variable. This set of signals can be used to define scores for the assessment of the coupling between systemic and brain hemodynamic variables. In essence a correction for SaO_2_ is provided by eliminating its contribution in the observed NIRS signal, which makes the residual component suited for the assessment of CAR even when changes in SaO_2_ are present.

#### Mathematical models

Several mathematical models have been used to assess CAR (37, 38). All of these methodologies try to quantify the relationship between ABP and CBF. These scores are then used to assess the status of the CAR mechanisms in the infants. These models can be divided in 2 main groups, linear and non-linear. The linear models can be further segmented in time-domain, frequency-domain, and non-stationary methods and are discussed below. However, visual inspection of the CAR curve indicates that the CAR mechanism should be nonlinear. Taking this into account several groups have studied CAR using nonlinear models with transcranial Doppler as a surrogate for CBF. However, clinical studies using nonlinear models for CAR assessment in neonates, when NIRS is used as a surrogate for CBF, are not identified during this systematic research.

##### Time domain

**Figure 4:**
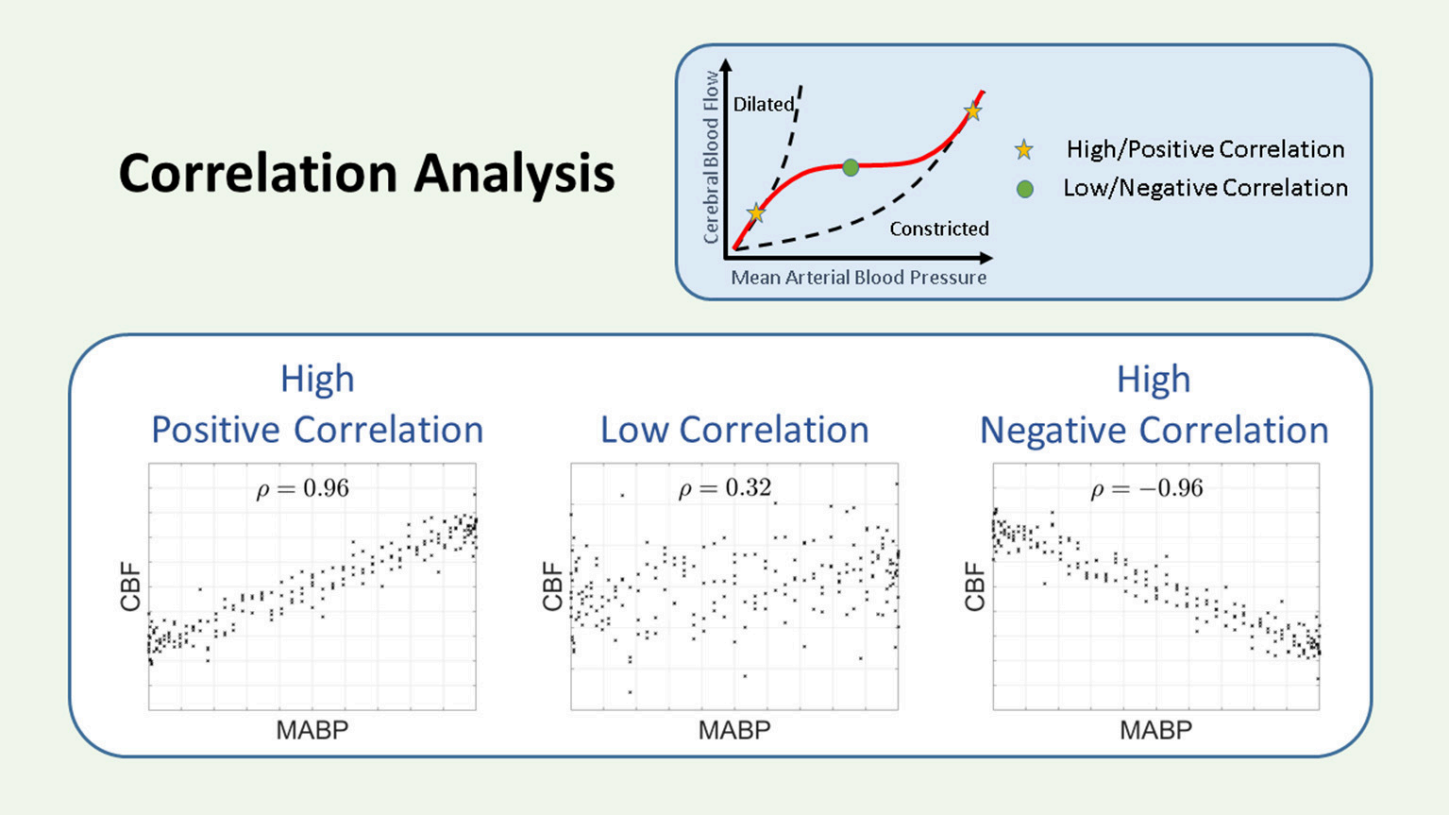
Schematic representation of the correlation. The COR coefficient is a measurement of the linear relationship between two variables. A large and positive COR coefficient indicates impaired CAR, while a small or negative COR coefficient indicates intact CAR. CAR, cerebral autoregulation; CBF, cerebral blood flow; COR, correlation; MABP, mean arterial blood pressure; ρ, correlation coefficient.

Among the temporal analysis of CAR, correlation (COR) and linear regression are most commonly used. Correlations between the NIRS-derived cerebral oxygenation and ABP measurement are often used to determine flow-pressure CAR. A graphical representation for the COR method can be seen in Figure 4. Different study groups use different correlation methods. Correlations can be carried out in predefined time epochs with or without a predefined threshold (22, 29–31, 39–42). In patients with CHD, a moving correlation coefficient method has been proposed by Brady et al. and named cerebral oximetry index (COx) (43). In preterm infants, COx was used to study the effect of dopamine on CAR (44, 45). A comparable method is used in patients with HIE using HVx (hemoglobin volume index), defining an optimal blood pressure MAP_opt_ from which deviations are correlated with outcome (46–50). Smith et al. used HVx in patients with CHD to study CAR during cardiopulmonary bypass (51). A similar moving correlation coefficient but with the use of Doppler was previously described in adults by Czosnyka et al. (52).

Additionally, some groups have explored the use of linear regression for the analysis of CAR. Tyszczuk et al. used COR coefficient and multiple linear regression to study CBF, MABP and other variables. However, no quantitative measurement of CAR was attempted (19). In contrast, Munro et al used linear regression in order to quantify the relationship between MABP and CBF. Based on this analysis they constructed curves for CAR and concluded that a breakpoint exists at 30 mmHg (53).

##### Frequency domain

Frequency domain analysis explores the relation between 2 signals in specific frequency bands. A major advantage is that it considers the fact that CAR may be composed of responses with different temporal properties, thereby taking into account the effect of time lag between changes in ABP and cerebral NIRS. Determination of the frequencies of interest is based on observed spontaneous oscillations and the cerebrovascular transit time and has an impact on the sample frequency. Different parameter choices like the length of the epochs, the frequency band and others will have an important influence on the calculation as described by Caicedo et al. (23).

Transfer Function (TF) analysis involves the computation of the signal power spectral density (PSD). The PSD of a signal is a representation of how much each particular frequency component contributes to form a given signal. In other terms, each measured signal can be considered as a sum of sinusoids with different frequencies, amplitudes and phases, the PSD represents how much power of each frequency is contained in the measured signal. Different methods exist to compute PSD. The most common is based on the Welch averaged periodogram (54). By measuring the PSD of the ABP and CBF, and their cross-power spectral density (CPSD), the coherence, gain and phase can be computed.

###### Coherence:

**Figure 5:**
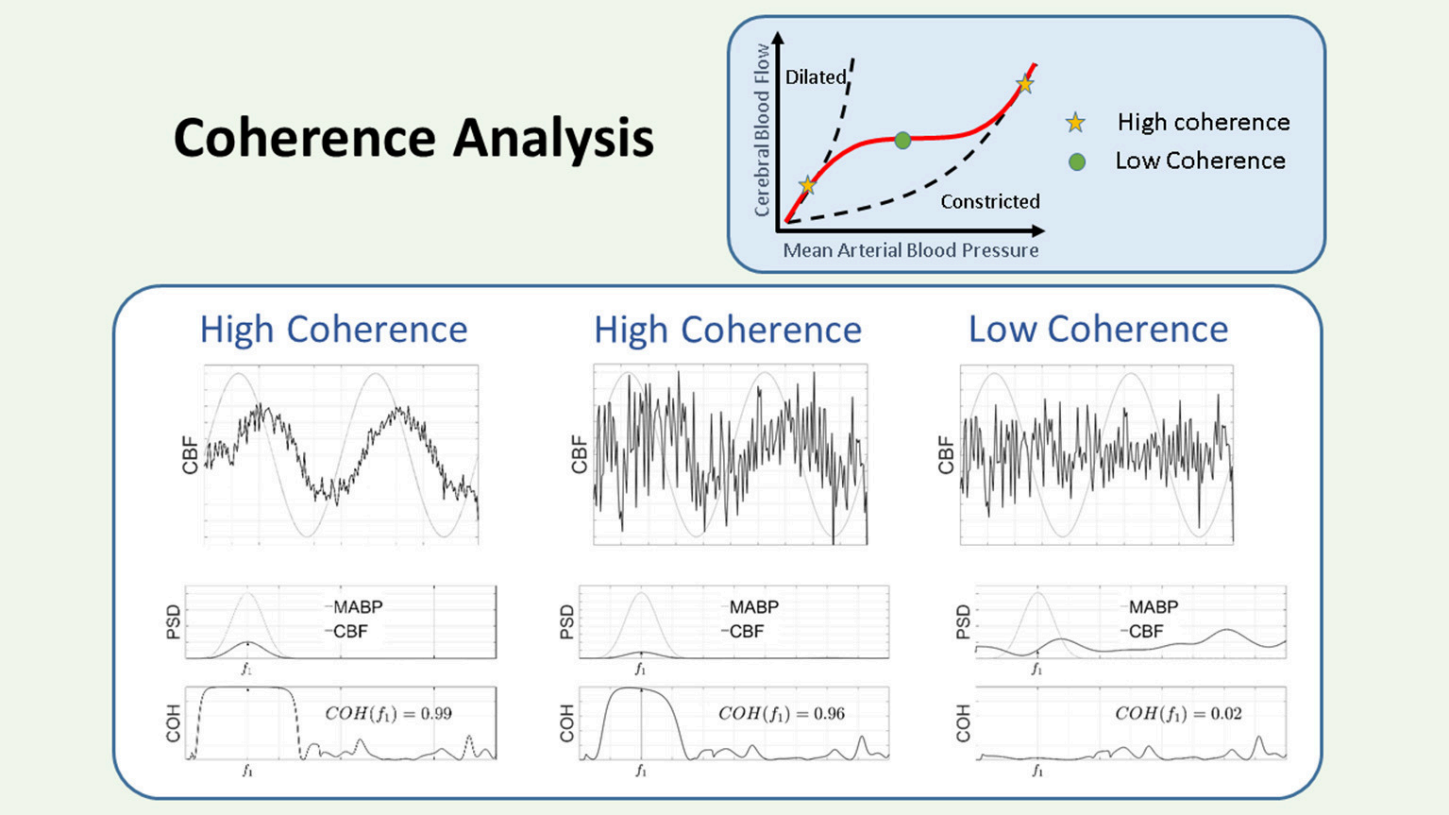
Schematic representation of the coherence. The COH is a measure of the linear dependencies between 2 signals. In the figure there are three panels, corresponding to three different conditions for the relation between the input: MABP (gray line), and the output: CBF (black line). Each panel is divided in an upper figure, representing the time course of the signals, a middle figure, representing their PSD, and a lower figure, representing the COH values in the region of interest. Since the input is sinusoidal, we consider the COH value as the value provided in the plot at that specified frequency. In the left panel, the output is contaminated with some noise, however it can be seen that the COH value is large, since the output contains a sinusoid of the same frequency as the input. In the middle panel, the sinusoid has been reduced in amplitude and more noise has been added to the output signal, however, as observed in the figure, the COH value is still large, since the output contains a sinusoid at that specified frequency. In the right panel, no sinusoid has been added to the output and only noise is considered for the computation of the COH. In this case it can be seen that the COH at the specified frequency is low. Within the framework of CAR a low COH value represents intact CAR, while a large COH is associated to impaired CAR. CAR, cerebral autoregulation; CBF, cerebral blood flow; COH, coherence; MABP, mean arterial blood pressure; PSD, power spectral density.

The coherence (COH) function describes the linearity of the relation between two signals in the frequency domain. In the framework of system theory, COH can be used as a measure to indicate if a linear relationship exists between the input and output of the system, which within the context of CAR assessment uses measurements of ABP as input, and NIRS-derived measurements of CBF as output, considering the CAR mechanism as the system to be identified. COH can be seen as the frequency based analog of COR in time. High COH values can be anticipated when CAR is impaired and a threshold for high COH can be defined or calculated according to the length of the windows. COH is used in different studies, with or without correction for SaO_2_ in specific time frames of 10–15 min or longer (40, 55–60). A graphical representation of the COH method is presented in Figure 5.

###### Gain:

**Figure 6:**
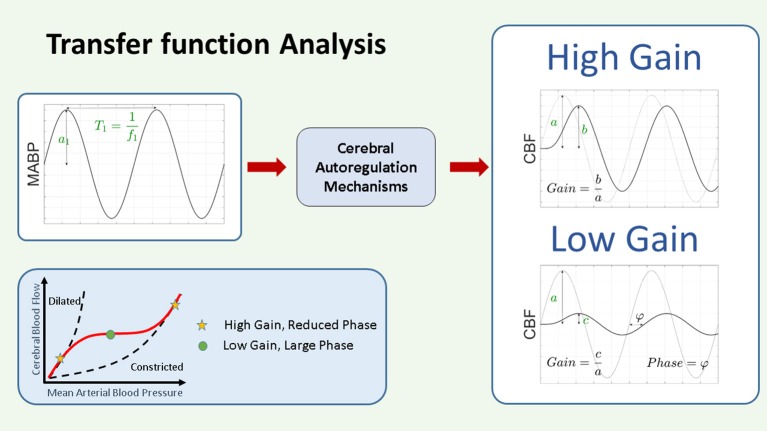
Schematic representation of transfer function gain and phase. In the framework of CAR as a system theory, the changes in ABP are considered as input and the changes in NIRS derived CBF as output. The CAR mechanisms are thus considered as a system that will be modeled by its TF. The TF analysis provides two outputs, the gain and the phase. The gain represents the magnitude of the relationship between the variables in the frequency domain, while the phase represents their relative shift in time. Assuming we have as input a pure sinusoidal change in MABP, as indicated in the figure, with this approach we will have a pure sinusoidal change in CBF at the same frequency. In this case the gain at that particular frequency can be interpreted as the ratio between the amplitude of the change in CBF and the change in MABP. A large gain indicates that the change in MABP produces a large change in CBF, while a small gain will indicate that the change in MABP will produce only a small change in CBF. On the other hand, the phase represents the time shift between the two signals. This time shift can be positive or negative. In the context of CAR analysis, a large gain and reduced phase indicate impaired CAR mechanism, while a low gain and large phase indicate intact CAR. CAR, cerebral autoregulation; CBF, cerebral blood flow; (M)ABP, mean arterial blood pressure.

TF analysis can be used to study the linear relation between physiological signals. In the framework of system theory and CAR, a TF uses measurements of ABP as input variables and NIRS-derived CBF as output. In this way, when using a TF, a model for the spectral behavior of the CAR mechanism can be found. This process assumes that CAR can be described by a linear, stationary system. The TF of a system can provide two features: the TF gain and the TF phase. TF gain represents the relationship in magnitude between the input and the output, while TF phase represents their temporal relation (time-shifts). A graphical representation of TF gain can be seen in Figure 6. Within the context of CAR, TF gain reflects the change in HbD/rScO_2_ caused by 1 mmHg change in ABP. The TF gain provides a measure of the magnitude of pressure passivity. A low TF gain would indicate that although CAR was not perfect, at least the magnitude of changes in CBF was small or moderate. This reflects the hypothesis that the plateau of the CAR curve is not flat but increases moderately (61). Similarly, a high TF gain would indicate that even moderate changes in ABP were associated with large changes in CBF. TF gain values have been analyzed over the complete set of measurements (62, 63) or only within coherent epochs (21, 60, 64, 66–68). TF analysis with logarithmic transformation of the gain coefficient to provide the amplitude of the dampening response is described by Vesoulis and measured in decibels (dB) where 0 dB represents no transformation and-10 dB represents a 10-fold reduction in power (69).

###### Phase:

In simple terms, the TF phase represents the time lag between the two signals. When using TF phase as a measure for CAR capacity, a high gain with a reduced phase has been associated to impaired autoregulation (60).

##### Non-stationary methods

Classical methods for the assessment of CAR, such as COR, COH, and TF gain, are often based on the assumption that changes in ABP and cerebral hemodynamics are stationary, i.e., assuming the statistical properties of these signals do not change with time. However, under pathophysiological conditions, but also during stable situations, ABP and CBF can behave non-stationary. Also, time information is averaged out by using TF gain. Thus, other methods are needed to describe the non-stationary aspects of CAR.

###### Continuous wavelet transform:

The CWT can be used to construct a time-frequency representation of a signal with a very good tradeoff between time and frequency resolution. It makes no assumption about the stationarity of input signals. Therefore, spectral analysis using wavelets provides a mathematical framework for the analysis of nonstationary effects in cerebral hemodynamics, thus overcoming the restrictions intrinsic to earlier methods (25). Tian et al. used rScO_2_ and described the setting of the neurovascular unit of the group of Chalak, together with EEG measurements (25). Tachtsidis et al. used HbO_2_ measurements, obtained with a multichannel NIRS device, to analyze the relationship between systemic variables and brain hemodynamics in ECMO patients (24). Using CWT several features or scores can be extracted, for instance compared to ordinary cross-correlations, Wavelet Cross-Correlation (WCC) is also a measure of similarity between two time series, but localized in frequencies, and taking into account the non-stationary nature of physiological measurements.

###### Bi-variate phase rectified signal averaging:

Bi-variate phase rectified signal averaging (BPRSA) is a method that is used in order to describe the response of a signal to changes in other signals. BPRSA identifies some points of interest in one signal, called the anchor points. These points are traditionally defined as increments or decrements of the signal. Then a segment around each anchor point is extracted from the other signal. All the segments are aligned with the anchor point positions in the middle, and averaged in order to produce the BPRSA response. Since the signal has been averaged over a large amount of segments, the influence of the non-stationarities as well as the noise on the estimation of the BPRSA curve is reduced. The BPRSA curve then represents how one signal reacts to increments or decrements of another variable, which can be of value for the study of the regulatory mechanism. In the context of CAR, Caicedo et al. used increments in MABP and HR as anchor points to study their effect on rScO_2_ and by studying the BPRSA curve they hypothesized that it was possible to assess CAR. They found differences in the obtained BPRSA curves between control subjects and subjects with an IVH (71).

###### Bivariate autoregressive coherence:

COH analysis, as presented before, is a very useful tool to detect linear dependencies among signals. However, in the case of third signals influencing both measurements, or in case that the interest lies in identifying the direction of influence, COH analysis is not the adequate tool. For this reason Riera et al. have used the bivariate autoregressive coherence (BiAR-COH). In short, BiAR-COH is able to produce a more reliable estimation of the linear dependencies among the signals, and indicate the directionality of the coupling. In order to do this, BiAR-COH first models the ABP as well as the TOI as follows: $\text{ABP}={\text{ABP}}_{{\text{ABP}}^{-}}+{\text{ABP}}_{{\text{TOI}}^{-}},\text{TOI}={\text{TOI}}_{{\text{TOI}}^{-}}+{\text{TOI}}_{{\text{ABP}}^{-}}$. When computing the COH assuming that ABP leads the changes in TOI, COH_ABP → TOI_, it makes more sense to use the component of TOI that is affected by ABP, TOI_ABP−_, instead of using the original ABP and TOI measurements. In this way it can be evaluated if changes in TOI can be predicted linearly by changes in ABP. BiAR-COH is computed as a ratio between the cross-power spectrum of $\text{TO}{\text{I}}_{\text{AB}{\text{P}}^{-}}$, and it is normalized using the sum of this cross-power spectrum and the power spectrum of $\text{AB}{\text{P}}_{\text{AB}{\text{P}}^{-}}$. Riera et al. found that this method has a good prognostic value to identify neonates at risk of brain hypoperfusion and adverse outcomes (72). Furthermore, they proposed the PDC as a valuable complementary analysis to BiAR-COH (34).

###### Corrections for variability in ABP:

One of the main drawbacks of different models for the assessment of CAR is that they require large changes in ABP and CBF in order to produce reliable scores. In long recordings it is possible to encounter these large changes. However, changes in ABP are not regular and thus non-stationary. Hahn et al. has indicated that weighting measurements with large in favor of those with small variations in ABP, increases the precision in the assessment of CAR (66, 67). Also other groups have taken the ABP variability into account for the assessment of CAR (73).

### Definition of impaired autoregulation

CAR is defined as a stable CBF during changes in CPP.

The CAR curve describes a plateau with a stable CBF between a lowest (minimum autoregulation pressure) and a highest (maximum autoregulation pressure) ABP. When the ABP decreases further, an almost linear relation between ABP and CBF is described possibly leading to ischemic lesions, while a further increase in ABP at the end of the plateau will give a linear increase in CBF leading to an overflow with possible IVH as a consequence. The plateau is not flat but slightly increasing explaining the low but significant gain during periods of autoregulation (61). The CAR curve can change in form by changes in CO_2_, PO_2_, acidosis, NO and other parameters causing vasodilation or vasoconstriction.

A loss of CAR means that the patient is -at that specific moment- outside the plateau of the curve. In contrast with earlier visions, recent observations suggest that this can change in individual patients according to changing parameters like ABP, CO_2_, sepsis, … Therefore, increasingly, studies describe the percentage of time of impaired CAR. However, it is important to notice that a loss of CAR does not necessarily means that the patient will develop complications, but is regarded as a higher risk for complications. Consequently, the clinician will aim for a stable CAR during treatment, within possible limits.

The definition of impaired CAR requires the definition of parameters that characterize where the individuals are located within the CAR curve or a specific cut-off point. Depending on the mathematical model used to study CAR, definitions can change. CAR can be seen as an on-off phenomenon. Different authors assess this phenomenon by identifying when the changes in ABP are linearly related to the changes in CBF. This is done by indicating when there is maximum COH, or COH above a certain threshold (20, 21, 59). However, others denote impaired CAR as a curve with a large slope in the autoregulation plateau and changes in the infliction points (53). Other groups use the change in CBF divided by the change in ABP or the autoregulation index (ARI) of static autoregulation (15). A wide range of definitions exist and thus, different ways of describing the autoregulation curve and its borders are used.

As mentioned before, some methods make use of thresholds in order to identify the instants of time when CAR is impaired. Different thresholds have been described using mathematical models, adult studies, animal studies, Monte Carlo simulations or visual inspection of data. Apart from cut-off values for COH and COR, also expressed as critical value score (CVS) (20, 40, 55, 57, 59), the threshold can also be expressed as amount of pressure passivity per total study time or per number of epochs. CPRT (critical percentage of recording time) (40, 57) and pressure passivity index (PPI) of COR,COH or TF gain is used by different groups (41, 57, 59, 68). The identifications of the lower infliction point is estimated by defining a lower limit of pressure autoregulation (LLA) by Brady et al. (43). The ABP associated with the most negative COR coefficient (less impaired CAR) is defined as MAP_opt_ in HIE studies (46–50).

### Impaired cerebral autoregulation and study outcome

Most studies in CAR try to relate the scores used for the assessment of CAR with clinical outcome. We will concisely present the different study outcomes for the different patient groups earlier described.

#### Preterms during the transitional period

This is the main research area for CAR in neonates and 25 articles were identified describing both short and long term outcome parameters. Starting in 2000, Tsuji described impaired CAR in 53% of ventilated preterms with a strong relationship between this impairment and severe IVH or periventricular leukomalacia (PVL) (20). The association between IVH and impaired CAR was confirmed by different groups (34, 42, 64, 69, 71–73), but not by others (30, 44, 63, 66).

A good correlation was described between impaired CAR and sickest infants, hypotension, and a higher CRIB-score. Different studies described the relation between low ABP and impaired CAR in preterms. Munro et al. described a cut-off value at 30 mmHg (53). Soul et al found a good correlation with hypotension, defined as less than the 10th percentile for PMA and postnatal age (59). They did not find a good correlation with blood pressure variability. Also, Hahn et al. described an association between impaired CAR and hypotension (defined as mean ABP in mmHg minus GA in weeks), but not with blood pressure variability (66). Gilmore et al. and Wong et al. described a correlation with lower ABP although no cut-off value was provided (21, 44).

Regarding long term outcome, impaired CAR was associated with a higher mortality and worse neurodevelopmental outcome by different groups (21, 34, 44, 59, 60, 63, 72). All studies were retrospective and no controlled prospective studies were performed.

#### Neonates receiving medication/treatment

Studies have explored whether treatment with volume/inotropes influences CAR. Treatment with dopamine is associated with impaired autoregulation in 4 studies (41, 45, 53, 74).

Neonatal effects on CAR of maternal drug treatment during the last days of pregnancy have been investigated (58, 62). Also, effects of certain postnatal drug treatments on CAR are described: surfactant administration in RDS (75), patent ductus arteriosus (PDA) treatment with indomethacin and/or surgery (41).

Extracorporeal membrane oxygenation (ECMO) treatment also disrupts CAR in neonates. The mechanisms are multifactorial, including heparinisation, as well as hemodynamic instability. In this context, it has been found that during low ECMO flow values, CAR is impaired in neonates undergoing ECMO weaning (24). In this study was also shown that the right hemisphere is more susceptible to disrupted CAR.

#### Patients with CHD

A single study has identified impaired CAR in 24 neonates with CHD in the preoperative phase. An association with lack of sedative medication, low hemoglobin, low mean ABP and greater FTOE was found (76). The group of Brady et al. has described an association with both hypotension and impairment of CAR during CPB. A cut-off value of 42±7 mmHg in pediatric patients undergoing cardiac surgery was described (43). However, hypothermia was identified as a confounding factor since ABP, temperature and CAR were collinear in a cohort studied later (51). In the early postoperative phase after CPB, higher end-tidal CO_2_, higher mean ABP variability but not lower ABP increased the odds of impaired CAR (55).

#### Patients with HIE treated with therapeutic hypothermia

A selected patient group to study CAR are infants with HIE after a perinatal ischemic event. Therapeutic hypothermia decreases the metabolic rate of the brain to preserve the remaining brain tissue as much as possible (78). By defining the optimal MAP (MAP_opt_) as the MAP where CAR is most robust, impaired CAR is identified in % of time, maximum deviation from and area under the curve below MAP_opt_. Retrospective studies where ABP measurements were set against this MAP_opt_ during hypothermia, rewarming and after the therapy showed an association between the impaired CAR and cardiopulmonary injury, subjective and objective injury on magnetic resonance imaging (MRI) and long term outcome (46–50). Other studies using different definitions have correlated impaired CAR with lesions on MRI and outcome (25, 68).

## Discussion

In this manuscript we provide an extensive overview of the measurement of CAR in neonates using NIRS.

### Clinical framework of CAR measurement

Different studies have shown that it is possible to measure CAR in neonates. No clear evidence for the best clinical framework has been provided. This may be caused due to the lack of a gold standard for its assessment. The lack of standardization on preprocessing methods and mathematical models used, length of window of analysis and thresholds of impaired CAR hinders the reproducibility of studies that have related CAR assessment with clinical outcome.

One of the first questions arising concerns the sample frequency. According to the Nyquist theorem, the sampling frequency must be at least twice the highest intrinsic frequency in the signal. To measure CAR, spontaneous cardiovascular oscillations are used. Some studies use the intrinsic variations of ABP within the low to very low frequency (frequency ≥0.0067 Hz, which corresponds to oscillations of 150 s) which represent the composite influence of autonomic, myogenic and cellular control mechanisms (79). Others use oscillations in the higher frequency range. The oscillations with a frequency component around 0.1 Hz are related to the Mayer waves and result from an oscillation of the sympathetic vasomotor tone. Several theories have been proposed to explain the constancy of the Mayer waves frequency (80). To use these waves to measure CAR, sampling frequencies should be at least 0.2 Hz (f_s_ ≥ 0.2 Hz, 1 value every 5 s). By using a lower signal sample frequency, information about the autoregulatory capacity of the brain around these higher frequencies will be lost. Several groups avoid measuring CAR using this 0.1 Hz frequency since the sensitivity of NIRS to brain activation is diminished by these physiological fluctuations that arises from scalp, skull and brain. These systemic changes may result in false positives by mimicking the brain hemodynamic response or result in false negatives by attenuating it (81). However, the question remains at which frequencies CAR can be studied best and which influences are measured.

Soul et al. and Bassan et al. were the first to measure bedside during days but often this involved a fellow being constantly present to make corrections in case of movement artifacts (55, 59). Therefore, the signal preprocessing, specifically artifact detection and removal, still needed further development in order to provide a reliable solution for the bedside monitoring of CAR. It has been shown that when assessing CAR, small artifacts can be easily truncated or interpolated (82). However, if large artifacts are present and large interpolations are made, the question arises if the results represent the real mechanisms under study.

In this context some efforts have been made in order to evaluate whether is possible to use some technologies that are more robust for CAR assessment. Caicedo et al, have shown that TOI and rScO_2_, can be used instead of HbD and HbT for the study of CAR. TOI and rScO_2_, derived from spatially resolved spectroscopy, are less prone to artifact movements than HbD and HbT (22).

### Comparisons between mathematical models

Few mathematical models are validated in animal studies. The COH and TF gain method to measure CAR is validated in piglets (83). A good correlation with TF gain was found when the COH was larger than 0.47. However, TF gain or COH alone were less optimal parameters, suggesting both parameters need to be used together to obtain a good assessment of CAR. The COx has been validated in piglets with hypotension (84) and correlates with transcranial Doppler-derived measurements of flow-pressure CAR in adult patients (43). Eriksen et al. compared COx (time domain) vs. COH and TF gain (frequency domain). They reported that time-domain analysis appeared more robust compared with COH function analysis. TF gain also increases when ABP and OI are in counter phase (67). This can be solved by including TF phase values, which provide an indication of the temporal shift between the signals in the frequencies of interest. One of the main drawbacks of the TF gain model is that it requires the presence of large variations, in a large range of frequencies, in MABP and CBF, in order to obtain reliable estimations of the spectral content of the signals. On the other hand, in the COx model, as with other COR models, the phase shift between the signals is not taken into account. These analyses assume that changes in ABP are immediately reflected in changes in CBF. Also, both COH and TF gain and COx models assume linearity, while this is not always true in biological signals. More studies comparing COH and TF gain vs. COx will elucidate the value of each model.

Another important issue concerns the precision of different details concerning the mathematical models. A comparison between selected details was published by Caicedo et al. The epoch length (segment on which the scores are calculated), overlapping percentage (different parameters in the COR and the frequency based methods), and sub-window length (segment used in the Welch method) all influenced importantly the final scores (23). This has been noticed by the Cerebral Autoregulation Network (CARNET, http://www.car-net.org/), which reported that TF gain and phase, which was used for CAR assessment, using ABP as input and transcranial Doppler measurements as output, change significantly between different centers. This was mainly due to the differences in preprocessing, selection of parameters for the estimation of PSD, among others (85). This phenomenon is also noted in the included studies causing difficulties in comparing study methods, definition of impaired CAR and outcome.

To overcome the non-stationary nature of biological signals, Chalak et al. focused on what they call the neurovascular unit approach, which involves the continuous monitoring of different modalities to identify potential mechanisms of dysfunctional CBF regulation by using the CWT. This model, compared to more traditional models, has the advantage to allow identification of the spectral content of the signal, together with the changes in time. Since CAR is a dynamic process, CWT seems to be more suited for its analysis. This strategy is proposed as a neuromonitoring tool in HIE to determine dynamic CAR (25, 86).

### Study outcome in CAR research

From a physiological point of view, impaired CAR is not by definition associated with pathology. Theoretically, infants with less adapted mechanisms to overcome the change in ABP are at risk for complications and can possible be identified by using CAR monitoring. If impaired CAR is identified, not all patients will develop complications.

Both short and long term outcome parameters are used in the studies described in this review. In general, the incidence of IVH is the outcome variable most frequently correlated with impaired CAR in preterm infants. Multiple animal and clinical studies have demonstrated that disturbed CBF is associated with IVH and PVL (1). This is reflected in studies where impaired CAR is associated with IVH using different mathematical methods (20, 42, 64, 69, 71–73). Alderliesten et al described a detailed time frame with impaired CAR and hyperperfusion before the occurrence of IVH. Other possible pathophysiological mechanisms are low SVC flow (34, 72) or low cardiac output (9, 19, 87). However, this association of IVH and impaired CAR is not confirmed in other studies (41, 44, 59). It is unknown whether the used method lacks precision or that in the subgroup studied, the impaired CAR did not necessarily provoke brain injury or other complications.

Correlation between impaired CAR and long term outcome is difficult due to other confounders (i.e., PMA, bronchopulmonary dysplasia, infection, medication and parental factors). This might be the reason why correlation with long term outcome is weak or absent.

CAR assessment during pharmacodynamic research is of special interest, since many drugs used in neonatal care are prescribed off-label. Identification of the drugs hemodynamic impact, both central and peripheral, is of utmost importance. Impairment of CAR after dopamine therapy is described in 4 previously mentioned studies. Also, in an extensive meta-analysis, increases in CBF following dopamine infusion were greater in hypotensive than normotensive preterm infants suggesting that dopamine does not exert a selective vasodilatory effect in the cerebral circulation and that the ABP of infants treated for hypotension was below the lower elbow of the CAR curve (88). However, impaired CAR might have developed by other confounders in this small patient group. A placebo-controlled randomized trial with dopamine in extremely premature infants is currently recruiting, to elucidate the effect of dopamine on long term outcome (89). A large patient group with measurements of invasive ABP and cerebral oxygenation will be available to study CAR during hypotension and treatment with dopamine or placebo.

### Limitations and future research

Due to the very heterogeneous study methods and outcome parameters, a meta-analysis was not performed. Due to small patient groups, corrections for other known variables defining long term outcome are not performed in most of the studies. At this stage, prospective interventional studies are lacking in all research areas.

After the identification of the patient at risk, continuous bedside CAR measurement can improve knowledge about cerebral pharmacodynamics of frequently used medication (sedatives-inotropes) and cerebral effects of specific therapies. However, most CAR studies are done offline, and at present, offline analysis is the standard. Online analysis to guide care, with bedside treatment adaptation if CAR is failing, may be possible in the future. In this way, physiology will be displayed on the multimodal monitor, hand in hand with the clinical examination.

A limitation for the continuous monitoring of CAR is the presence of an arterial line to monitor ABP invasively. A promising technique is to use heart rate instead of ABP to describe CAR. Describing the functional activation of the brain, heart rate is used as a surrogate marker for neurogenic activity (35, 60, 71, 90, 91). Da Costa et al. used moving correlation between heart rate and TOI to determine optimal ABP (92). Cardiac output is another possible variable that can be used as a surrogate for APB (9, 93).

In the future, multimodal monitoring databases of NICU-patients of multiple centers, together with identical outcome parameters are needed to compare different mathematical models and make progress in this field. To achieve this, easy, cheap, user-friendly NIRS-equipment with validated neonatal sensors is necessary.

## Conclusion

NIRS derived CAR measurement is an important research tool to improve knowledge about central hemodynamic fluctuations during the transitional period, cerebral pharmacodynamics of frequently used medication (sedatives-inotropes) and cerebral effects of specific therapies in neonatology. Uniformity regarding measurement techniques and mathematical models is needed. Multimodal monitoring databases of neonatal intensive care patients of multiple centers, together with identical outcome parameters are needed to compare different techniques and make progress in this field. Real-time bedside monitoring of CAR, together with conventional monitoring, seems a promising technique to improve individual patient care.

## Author contributions

LT screened the article titles and abstracts to determine whether they met the inclusion criteria. Then, LT reviewed full text articles to assess for eligibility. Any articles presenting doubts or inconsistencies were fully reviewed by GN and AC until a decision was reached on their inclusion. The first draft was made by LT, GN, and AC and all other authors contributed to the final article.

### Conflict of interest statement

The authors declare that the research was conducted in the absence of any commercial or financial relationships that could be construed as a potential conflict of interest.

## Abbreviations

ADC: apparent diffusion coefficient
ARI: Autoregulation index
AUC: area under the curve
BW: birth weight
BPRSA: bivariate phase rectified signal averaging
BPV: blood pressure variability
CAR: cerebral autoregulation
CBF: cerebral blood flow
(c)FTOE: cerebral fractional tissue oxygen extraction
CHD: congenital heart disease
CO_2_: carbon dioxide
COH_ST_: standardized COH
COR: correlation
COx: cerebral oximetry index
CPB: cardiopulmonary bypass
CPC: common part method
CPRT: critical percentage of recording time
CPSD: cross-power spectral density
CRIB: clinical risk index for babies
CVS: critical value score
CWT: continuous wavelet transform
dB: decibel
DOL: day of life
ECMO: extracorporeal membrane oxygenation
GA: gestational age
HbD/Hb_Diff_, HbO_2_-HHb, hemoglobin difference: cerebral intravascular oxygenation
HHb: deoxygenated hemoglobin
HbO_2_: oxygenated hemoglobin
HIE: hypoxic-ischemic encephalopathy
HVx: hemoglobin volume index
Hz: Hertz
INSURE, intubation: surfactant administration and immediate extubation
LISA: less invasive surfactant administration
LLA: lower limit of pressure autoregulation
(M)A(B)P: (mean) arterial (blood) pressure
Max: maximum
MAP_opt_: optimal mean arterial blood pressure
MDI: mental developmental index
MRI: magnetic resonance imaging
NEC: necrotizing enterocolitis
NIRS: near-infrared spectroscopy
ObSP: oblique subspace projections
PACOH: partial coherence
(p)BiAR-COH: (patient average) bivariate autoregressive spectral coherence
(p)COH: (patient average) (spectral) coherence
PDA: patent ductus arteriosus
PDI: psychomotor developmental index
(P)IVH: (peri-)intraventricular hemorrhage
PMA: postmenstrual age
(p)PDC: (patient averaged) partial directed coherence method
PPI: pressure passivity index
PVL: periventricular leukomalacia
PSD: power spectral density
r_c_SO_2_/SctO_2_/crSO_2_/rSO_2_/ScO_2_/rSco_2_/rSo_2_: regional cerebral tissue oxygen saturation
RDS: respiratory distress syndrome
rTHb: relative total tissue hemoglobin
SD: standard deviation
SVC: superior vena cava
tHb/HbT/Hb_Total_, total hemoglobin: cerebral Hb volume
TF: transfer function
(T)OI: (tissue) oxygenation index
WCC: wavelet cross correlation
WR-OBP: wavelet regression and oblique subspace projections.

## References

[1] Volpe JJ. Brain injury in premature infants: a complex amalgam of destructive and developmental disturbances. Lancet Neurol. (2009) 8:110–24. 10.1016/S1474-4422(08)70294-1 19081519 PMC2707149

[2] Volpe JJ. Intraventricular hemorrhage and brain injury in the premature infant. Neuropathology and pathogenesis. Clin Perinatol. (1989) 16:361–86. 2663307

[3] Garner RS Burchfield DJ. Treatment of presumed hypotension in very low birthweight neonates: effects on regional cerebral oxygenation. Arch Dis Child Fetal Neonatal Ed. (2013) 98:F117–21. 10.1136/archdischild-2011-301488 22782995

[4] Smits A Thewissen L Dereymaeker A Dempsey E Caicedo A Naulaers G. The use of hemodynamic and cerebral monitoring to study pharmacodynamics in neonates. Curr Pharm Des. (2017) 23:5955–63. 10.2174/1381612823666170918124419 28925890

[5] Lassen NA. Cerebral blood flow and oxygen consumption in man. Physiol Rev. (1959) 39:183–238. 10.1152/physrev.1959.39.2.183 13645234

[6] Harper AM Jennett S Cerebral Blood Flow and Metabolism. Manchester, UK: Manchester University Press (1990).

[7] Panerai RB Deverson ST Mahony P Hayes P Evans DH. Effects of CO_2_ on dynamic cerebral autoregulation measurement. Physiol Meas. (1999) 20:265–75. 10.1088/0967-3334/20/3/304 10475580

[8] Huneau C Benali H Chabriat H. Investigating human neurovascular coupling using functional neuroimaging: a critical review of dynamic models. Front Neurosci. (2015) 9:467. 10.3389/fnins.2015.00467 26733782 PMC4683196

[9] Weindling AM, Kissack CM. Blood pressure and tissue oxygenation in the newborn baby at risk of brain damage. Biol Neonate (2001) 79:241–5. 10.1159/000047099 11275659

[10] Greisen G. Autoregulation of cerebral blood flow in newborn babies. Early Hum Dev. (2005) 81:423–8. 10.1016/j.earlhumdev.2005.03.005 15935919

[11] Lou HC Lassen NA Friis-Hansen B. Impaired autoregulation of cerebral blood flow in the distressed newborn infant. J Pediatr. (1979) 94:118–21. 10.1016/S0022-3476(79)80373-X 758388

[12] Milligan DW. Failure of autoregulation and intraventricular haemorrhage in preterm infants. Lancet (1980) 1:896–8. 10.1016/S0140-6736(80)90836-3 6103257

[13] Pryds O Greisen G Lou H Friis-Hansen B. Heterogeneity of cerebral vasoreactivity in preterm infants supported by mechanical ventilation. J Pediatr. (1989) 115:638–45. 10.1016/S0022-3476(89)80301-4 2507767

[14] Volpe JJ. Brain injury in the premature infant: overview of clinical aspects, neuropathology, and pathogenesis. Semin Pediatr Neurol. (1998) 5:135–51. 10.1016/S1071-9091(98)80030-2 9777673

[15] Wagner BP Ammann RA Bachmann DC Born S Schibler A. Rapid assessment of cerebral autoregulation by near-infrared spectroscopy and a single dose of phenylephrine. Pediatr Res. (2011) 69:436–41. 10.1203/PDR.0b013e3182110177 21258266

[16] Panerai RB Kelsall AW Rennie JM Evans DH. Cerebral autoregulation dynamics in premature newborns. Stroke (1995) 26:74–80. 10.1161/01.STR.26.1.74 7839402

[17] Naulaers G Meyns B Miserez M Leunens V Van Huffel S Casaer P. Use of tissue oxygenation index and fractional tissue oxygen extraction as non-invasive parameters for cerebral oxygenation. A validation study in piglets. Neonatology (2007) 92:120–6. 10.1159/000101063 17377413

[18] Dix LM Van Bel F Lemmers PM Monitoring cerebral oxygenation in neonates: an update. Front Pediatr. (2017) 5:46 10.3389/fped.2017.00046 28352624 PMC5348638

[19] Tyszczuk L Meek J Elwell C Wyatt JS. Cerebral blood flow is independent of mean arterial blood pressure in preterm infants undergoing intensive care. Pediatrics (1998) 102:337–41. 10.1542/peds.102.2.337 9685435

[20] Tsuji M Saul JP Du Plessis A Eichenwald E Sobh J Crocker R. Cerebral intravascular oxygenation correlates with mean arterial pressure in critically ill premature infants. Pediatrics (2000) 106:625–32. 10.1542/peds.106.4.625 11015501

[21] Wong FY Leung TS Austin T Wilkinson M Meek JH Wyatt JS. Impaired autoregulation in preterm infants identified by using spatially resolved spectroscopy. Pediatrics (2008) 121:e604–11. 10.1542/peds.2007-1487 18250118

[22] Caicedo A De Smet D Naulaers G Ameye L Vanderhaegen J Lemmers P. Cerebral tissue oxygenation and regional oxygen saturation can be used to study cerebral autoregulation in prematurely born infants. Pediatr Res. (2011) 69:548–53. 10.1203/PDR.0b013e3182176d85 21364491

[23] Caicedo A Naulaers G Lemmers P Van Bel F Wolf M Van Huffel S. Detection of cerebral autoregulation by near-infrared spectroscopy in neonates: performance analysis of measurement methods. J Biomed Opt. (2012) 17:117003. 10.1117/1.JBO.17.11.117003 23117814

[24] Papademetriou MD Tachtsidis I Elliot MJ Hoskote A Elwell CE. Multichannel near infrared spectroscopy indicates regional variations in cerebral autoregulation in infants supported on extracorporeal membrane oxygenation. J Biomed Opt. (2012) 17:067008. 10.1117/1.JBO.17.6.067008 22734786

[25] Tian F Tarumi T Liu H Zhang R Chalak L. Wavelet coherence analysis of dynamic cerebral autoregulation in neonatal hypoxic-ischemic encephalopathy. Neuroimage Clin. (2016) 11:124–32. 10.1016/j.nicl.2016.01.020 26937380 PMC4753811

[26] Schat TE Van Der Laan ME Schurink M Hulscher JB Hulzebos CV Bos AF. Assessing cerebrovascular autoregulation in infants with necrotizing enterocolitis using near-infrared spectroscopy. Pediatr Res. (2016) 79:76–80. 10.1038/pr.2015.184 26383883

[27] Scholkmann F Spichtig S Muehlemann T Wolf M. How to detect and reduce movement artifacts in near-infrared imaging using moving standard deviation and spline interpolation. Physiol Meas. (2010) 31:649–62. 10.1088/0967-3334/31/5/004 20308772

[28] Ayaz H Izzetoglu M Shewokis PA Onaral B. Sliding-window motion artifact rejection for Functional Near-Infrared Spectroscopy. Conf Proc IEEE Eng Med Biol Soc. (2010) 2010:6567–70. 10.1109/IEMBS.2010.5627113 21096508

[29] Lemmers PM Toet M Van Schelven LJ Van Bel F. Cerebral oxygenation and cerebral oxygen extraction in the preterm infant: the impact of respiratory distress syndrome. Exp Brain Res. (2006) 173:458–67. 10.1007/s00221-006-0388-8 16506004

[30] Verhagen EA Hummel LA Bos AF Kooi EM. Near-infrared spectroscopy to detect absence of cerebrovascular autoregulation in preterm infants. Clin Neurophysiol. (2014) 125:47–52. 10.1016/j.clinph.2013.07.001 23973384

[31] Kooi EM Van Der Laan ME Verhagen EA Van Braeckel KN Bos AF Volume expansion does not alter cerebral tissue oxygen extraction in preterm infants with clinical signs of poor perfusion. Neonatology (2013) 103:308–14. 10.1159/000346383 23548640

[32] De Smet D Jacobs J Ameye L Vanderhaegen J Naulaers G Lemmers P. The partial coherence method for assessment of impaired cerebral autoregulation using near-infrared spectroscopy: potential and limitations. Adv Exp Med Biol. (2010) 662:219–24. 10.1007/978-1-4419-1241-1_31 20204795

[33] Baccala LA Sameshima K. Partial directed coherence: a new concept in neural structure determination. Biol Cybern. (2001) 84:463–74. 10.1007/PL00007990 11417058

[34] Riera J Cabanas F Serrano JJ Madero R Pellicer A. New developments in cerebral blood flow autoregulation analysis in preterm infants: a mechanistic approach. Pediatr Res. (2016) 79:460–5. 10.1038/pr.2015.231 26539666

[35] Caicedo A Alderliesten T Naulaers G Lemmers P Van Bel F Van Huffel S. A new framework for the assessment of cerebral hemodynamics regulation in neonates using NIRS. Adv Exp Med Biol. (2016) 876:501–9. 10.1007/978-1-4939-3023-4_63 26782251

[36] Caicedo A Varon C Hunyadi B Papademetriou M Tachtsidis I Van Huffel S. Decomposition of near-infrared spectroscopy signals using oblique subspace projections: applications in brain hemodynamic monitoring. Front Physiol. (2016) 7:515. 10.3389/fphys.2016.00515 27877133 PMC5099173

[37] Panerai RB. Assessment of cerebral pressure autoregulation in humans–a review of measurement methods. Physiol Meas. (1998) 19:305–38. 10.1088/0967-3334/19/3/001 9735883

[38] Kooi EMW Verhagen EA Elting JWJ Czosnyka M Austin T Wong FY. Measuring cerebrovascular autoregulation in preterm infants using near-infrared spectroscopy: an overview of the literature. Expert Rev Neurother. (2017) 17:801–18. 10.1080/14737175.2017.1346472 28639837

[39] Binder-Heschl C Urlesberger B Schwaberger B Koestenberger M Pichler G. Borderline hypotension: how does it influence cerebral regional tissue oxygenation in preterm infants? J Matern Fetal Neonatal Med. (2016) 29:2341–6. 10.3109/14767058.2015.1085020 26381128

[40] De Smet D Vanderhaegen J Naulaers G Van Huffel S. New measurements for assessment of impaired cerebral autoregulation using near-infrared spectroscopy. Adv Exp Med Biol. (2009) 645:273–8. 10.1007/978-0-387-85998-9_41 19227482

[41] Chock VY Ramamoorthy C Van Meurs KP. Cerebral autoregulation in neonates with a hemodynamically significant patent ductus arteriosus. J Pediatr. (2012) 160:936–42. 10.1016/j.jpeds.2011.11.054 22226574 PMC3335982

[42] Alderliesten T Lemmers PM Smarius JJ Van De Vosse RE Baerts W Van Bel F. Cerebral oxygenation, extraction, and autoregulation in very preterm infants who develop peri-intraventricular hemorrhage. J Pediatr. (2013) 162:698–704.e692. 10.1016/j.jpeds.2012.09.038 23140883

[43] Brady KM Mytar JO Lee JK Cameron DE Vricella LA Thompson WR. Monitoring cerebral blood flow pressure autoregulation in pediatric patients during cardiac surgery. Stroke (2010) 41:1957–62. 10.1161/STROKEAHA.109.575167 20651273 PMC5498798

[44] Gilmore MM Stone BS Shepard JA Czosnyka M Easley RB Brady KM. Relationship between cerebrovascular dysautoregulation and arterial blood pressure in the premature infant. J Perinatol. (2011) 31:722–9. 10.1038/jp.2011.17 21372795

[45] Eriksen VR Hahn GH Greisen G. Dopamine therapy is associated with impaired cerebral autoregulation in preterm infants. Acta Paediatr. (2014) 103:1221–6. 10.1111/apa.12817 25266994

[46] Lee JK Poretti A Perin J Huisman T Parkinson C Chavez-Valdez R. Optimizing cerebral autoregulation may decrease neonatal regional hypoxic-ischemic brain injury. Dev Neurosci. (2017) 39:248–56. 10.1159/000452833 27978510 PMC5474204

[47] Chavez-Valdez R O'connor M Perin J Reyes M Armstrong J Parkinson C. Sex-specific associations between cerebrovascular blood pressure autoregulation and cardiopulmonary injury in neonatal encephalopathy and therapeutic hypothermia. Pediatr Res. (2017) 81:759–66. 10.1038/pr.2017.23 28141793 PMC5561426

[48] Tekes A Poretti A Scheurkogel MM Huisman TA Howlett JA Alqahtani E. Apparent diffusion coefficient scalars correlate with near-infrared spectroscopy markers of cerebrovascular autoregulation in neonates cooled for perinatal hypoxic-ischemic injury. AJNR Am J Neuroradiol. (2015) 36:188–93. 10.3174/ajnr.A4083 25169927 PMC4359612

[49] Burton VJ Gerner G Cristofalo E Chung SE Jennings JM Parkinson C. A pilot cohort study of cerebral autoregulation and 2-year neurodevelopmental outcomes in neonates with hypoxic-ischemic encephalopathy who received therapeutic hypothermia. BMC Neurol. (2015) 15:209. 10.1186/s12883-015-0464-4 26486728 PMC4618147

[50] Howlett JA Northington FJ Gilmore MM Tekes A Huisman TA Parkinson C. Cerebrovascular autoregulation and neurologic injury in neonatal hypoxic-ischemic encephalopathy. Pediatr Res. (2013) 74:525–35. 10.1038/pr.2013.132 23942555 PMC3954983

[51] Smith B Vu E Kibler K Rusin C Easley RB Andropoulos D. Does hypothermia impair cerebrovascular autoregulation in neonates during cardiopulmonary bypass? Paediatr Anaesth. (2017) 27:905–10. 10.1111/pan.13194 28653463

[52] Czosnyka M Smielewski P Lavinio A Pickard JD Panerai R. An assessment of dynamic autoregulation from spontaneous fluctuations of cerebral blood flow velocity: a comparison of two models, index of autoregulation and mean flow index. Anesth Analg. (2008) 106:234–9, table of contents. 10.1213/01.ane.0000295802.89962.13 18165583

[53] Munro MJ Walker AM Barfield CP. Hypotensive extremely low birth weight infants have reduced cerebral blood flow. Pediatrics (2004) 114:1591–6. 10.1542/peds.2004-1073 15574619

[54] Welch P The use of fast Fourier transform for the estimation of power spectra: a method based on time averaging over short, modified periodograms. IEEE Tran. Audio Electroacoust. (1967) 15:70–3. 10.1109/TAU.1967.1161901

[55] Bassan H Gauvreau K Newburger JW Tsuji M Limperopoulos C Soul JS. Identification of pressure passive cerebral perfusion and its mediators after infant cardiac surgery. Pediatr Res. (2005) 57:35–41. 10.1203/01.PDR.0000147576.84092.F9 15531739

[56] Hahn GH Christensen KB Leung TS Greisen G. Precision of coherence analysis to detect cerebral autoregulation by near-infrared spectroscopy in preterm infants. J Biomed Opt. (2010) 15:037002. 10.1117/1.3426323 20615031

[57] Caicedo A De Smet D Vanderhaegen J Naulaers G Wolf M Lemmers P. Impaired cerebral autoregulation using near-infrared spectroscopy and its relation to clinical outcomes in premature infants. Adv Exp Med Biol. (2011) 701:233–9. 10.1007/978-1-4419-7756-4_31 21445792

[58] Baerts W Van Bel F Thewissen L Derks JB Lemmers PM. Tocolytic indomethacin: effects on neonatal haemodynamics and cerebral autoregulation in the preterm newborn. Arch Dis Child Fetal Neonatal Ed. (2013) 98: F419–23. 10.1136/archdischild-2012-302532 23482639

[59] Soul JS Hammer PE Tsuji M Saul JP Bassan H Limperopoulos C. Fluctuating pressure-passivity is common in the cerebral circulation of sick premature infants. Pediatr Res. (2007) 61:467–73. 10.1203/pdr.0b013e31803237f6 17515873

[60] Zhang Y Chan GS Tracy MB Lee QY Hinder M Savkin AV. Spectral analysis of systemic and cerebral cardiovascular variabilities in preterm infants: relationship with clinical risk index for babies (CRIB). Physiol Meas. (2011) 32:1913–28. 10.1088/0967-3334/32/12/003 22048689

[61] Greisen G. To autoregulate or not to autoregulate–that is no longer the question. Semin Pediatr Neurol. (2009) 16:207–15. 10.1016/j.spen.2009.09.002 19945655

[62] Caicedo A Thewissen L Naulaers G Lemmers P Van Bel F Van Huffel S. Effect of Maternal use of Labetalol on the Cerebral Autoregulation in Premature Infants. Adv Exp Med Biol. (2013) 789:105–11. 10.1007/978-1-4614-7411-1_15 23852483

[63] Caicedo A Naulaers G Wolf M Lemmers P Van Bel F Ameye L. Assessment of the myogenic and metabolic mechanism influence in cerebral autoregulation using near-infrared spectroscopy. Adv Exp Med Biol. (2012) 737:37–44. 10.1007/978-1-4614-1566-4_6 22259079

[64] O'leary H Gregas MC Limperopoulos C Zaretskaya I Bassan H Soul JS. Elevated cerebral pressure passivity is associated with prematurity-related intracranial hemorrhage. Pediatrics (2009) 124:302–9. 10.1542/peds.2008-2004 19564313 PMC4030537

[65] Morren G Naulaers G Lemmerling P Van Huffel S Casaer P Devlieger H. Quantitation of the concordance between cerebral intravascular oxygenation and mean arterial blood pressure for the detection of impaired autoregulation. Adv Exp Med Biol. (2003) 510:403–8. 10.1007/978-1-4615-0205-0_67 12580462

[66] Hahn GH Maroun LL Larsen N Hougaard DM Sorensen LC Lou HC. Cerebral autoregulation in the first day after preterm birth: no evidence of association with systemic inflammation. Pediatr Res. (2012) 71:253–60. 10.1038/pr.2011.46 22278187

[67] Eriksen VR Hahn GH Greisen G. Cerebral autoregulation in the preterm newborn using near-infrared spectroscopy: a comparison of time-domain and frequency-domain analyses. J Biomed Opt. (2015) 20:037009. 10.1117/1.JBO.20.3.037009 25806662

[68] Massaro AN Govindan RB Vezina G Chang T Andescavage NN Wang Y. Impaired cerebral autoregulation and brain injury in newborns with hypoxic-ischemic encephalopathy treated with hypothermia. J Neurophysiol. (2015) 114:818–24. 10.1152/jn.00353.2015 26063779 PMC4533061

[69] Vesoulis ZA Liao SM Trivedi SB Ters NE Mathur AM. A novel method for assessing cerebral autoregulation in preterm infants using transfer function analysis. Pediatr Res. (2016) 79:453–9. 10.1038/pr.2015.238 26571222 PMC4821724

[70] Stammwitz A Von Siebenthal K Bucher HU Wolf M. Can the assessment of spontaneous oscillations by near infrared spectrophotometry predict neurological outcome of preterm infants? Adv Exp Med Biol. (2016) 876:521–31. 10.1007/978-1-4939-3023-4_65 26782253

[71] Caicedo A Varon C Alderliesten T Lemmers P Van Bel F Naulaers G. Differences in the cerebral hemodynamics regulation mechanisms of premature infants with intra-ventricular hemorrhage assessed by means of phase rectified signal averaging. Conf Proc IEEE Eng Med Biol Soc. (2014) 2014:4208–11. 10.1109/EMBC.2014.6944552 25570920

[72] Riera J Cabanas F Serrano JJ Bravo MC Lopez-Ortego P Sanchez L. New time-frequency method for cerebral autoregulation in newborns: predictive capacity for clinical outcomes. J Pediatr. (2014) 165:897–902.e891. 10.1016/j.jpeds.2014.06.008 25039050

[73] Wong FY Silas R Hew S Samarasinghe T Walker AM. Cerebral oxygenation is highly sensitive to blood pressure variability in sick preterm infants. PLoS ONE (2012) 7:e43165. 10.1371/journal.pone.0043165 22905222 PMC3419198

[74] Alderliesten T Lemmers PM Van Haastert IC De Vries LS Bonestroo HJ Baerts W Hypotension in preterm neonates: low blood pressure alone does not affect neurodevelopmental outcome. J Pediatr. (2014) 164:986–91. 10.1016/j.jpeds.2013.12.042 24484771

[75] Li XF Cheng TT Guan RL Liang H Lu WN Zhang JH. Effects of different surfactant administrations on cerebral autoregulation in preterm infants with respiratory distress syndrome. J Huazhong Univ Sci Technolog Med Sci. (2016) 36:801–5. 10.1007/s11596-016-1665-9 27924521

[76] Votava-Smith JK Statile CJ Taylor MD King EC Pratt JM Nelson DP. Impaired cerebral autoregulation in preoperative newborn infants with congenital heart disease. J Thorac Cardiovasc Surg. (2017) 154:1038–44. 10.1016/j.jtcvs.2017.05.045 28634025

[77] Lee JK Poretti A Perin J Huisman T Parkinson C Chavez-Valdez R. Optimizing cerebral autoregulation may decrease neonatal regional hypoxic-ischemic brain injury. Dev Neurosci. (2017) 39:248–56. 27978510 10.1159/000452833 PMC5474204

[78] Drury PP Bennet L Gunn AJ. Mechanisms of hypothermic neuroprotection. Semin Fetal Neonatal Med. (2010) 15:287–92. 10.1016/j.siny.2010.05.005 20646974

[79] Vesoulis ZA Mathur AM. Cerebral autoregulation, brain injury, and the transitioning premature infant. Front Pediatr. (2017) 5:64. 10.3389/fped.2017.00064 28421173 PMC5377300

[80] Julien C. The enigma of mayer waves: facts and models. Cardiovasc Res. (2006) 70:12–21. 10.1016/j.cardiores.2005.11.008 16360130

[81] Yucel MA Selb J Aasted CM Lin PY Borsook D Becerra L. Mayer waves reduce the accuracy of estimated hemodynamic response functions in functional near-infrared spectroscopy. Biomed Opt Express (2016) 7:3078–88. 10.1364/BOE.7.003078 27570699 PMC4986815

[82] Caicedo A Van Huffel S. Weighted LS-SVM for function estimation applied to artifact removal in bio-signal processing. In: 2010 Annual International Conference of the IEEE Engineering in Medicine and Biology. Buenos Aires. (2010). p. 988–991. 21096987 10.1109/IEMBS.2010.5627628

[83] Hahn GH Heiring C Pryds O Greisen G. Applicability of near-infrared spectroscopy to measure cerebral autoregulation noninvasively in neonates: a validation study in piglets. Pediatr Res. (2011) 70:166–70. 10.1203/PDR.0b013e3182231d9e 21566541

[84] Brady KM Lee JK Kibler KK Smielewski P Czosnyka M Easley RB. Continuous time-domain analysis of cerebrovascular autoregulation using near-infrared spectroscopy. Stroke (2007) 38:2818–25. 10.1161/STROKEAHA.107.485706 17761921 PMC2377358

[85] Meel-Van Den Abeelen AS De Jong DL Lagro J Panerai RB Claassen JA. How measurement artifacts affect cerebral autoregulation outcomes: a technical note on transfer function analysis. Med Eng Phys. (2016) 38:490–7. 10.1016/j.medengphy.2016.02.001 26935320

[86] Chalak LF Tarumi T Zhang R The “neurovascular unit approach” to evaluate mechanisms of dysfunctional autoregulation in asphyxiated newborns in the era of hypothermia therapy. Early Hum Dev. (2014) 90:687–94. 10.1016/j.earlhumdev.2014.06.013 25062804 PMC4170014

[87] Wardle SP Yoxall CW Weindling AM. Determinants of cerebral fractional oxygen extraction using near infrared spectroscopy in preterm neonates. J Cereb Blood Flow Metab. (2000) 20:272–9. 10.1097/00004647-200002000-00008 10698064

[88] Sassano-Higgins S Friedlich P Seri I. A meta-analysis of dopamine use in hypotensive preterm infants: blood pressure and cerebral hemodynamics. J Perinatol. (2011) 31:647–55. 10.1038/jp.2011.2 21273985

[89] Dempsey EM Barrington KJ Marlow N O'donnell CP Miletin J Naulaers G. Management of hypotension in preterm infants (The HIP Trial): a randomised controlled trial of hypotension management in extremely low gestational age newborns. Neonatology (2014) 105:275–81. 10.1159/000357553 24576799

[90] Caicedo A Varon C Thewissen L Naulaers G Lemmers P Van Bel F. Influence of the maternal use of labetalol on the neurogenic mechanism for cerebral autoregulation assessed by means of NIRS. Adv Exp Med Biol. (2014) 812:173–9. 10.1007/978-1-4939-0620-8_23 24729230

[91] Mitra S Czosnyka M Smielewski P O'reilly H Brady K Austin T. Heart rate passivity of cerebral tissue oxygenation is associated with predictors of poor outcome in preterm infants. Acta Paediatr. (2014) 103:e374–82. 10.1111/apa.12696 24844816

[92] Da Costa CS Czosnyka M Smielewski P Mitra S Stevenson GN Austin T. Monitoring of Cerebrovascular Reactivity for Determination of Optimal Blood Pressure in Preterm Infants. J Pediatrics (2015) 167:86–91. 10.1016/j.jpeds.2015.03.041 25891381

[93] Noone MA Sellwood M Meek JH Wyatt JS. Postnatal adaptation of cerebral blood flow using near infrared spectroscopy in extremely preterm infants undergoing high-frequency oscillatory ventilation. Acta Paediatr. (2003) 92:1079–84. 10.1111/j.1651-2227.2003.tb02581.x 14599074

